# NAuRA: Genomic Tool to Identify Staphylococcal Enterotoxins in *Staphylococcus aureus* Strains Responsible for FoodBorne Outbreaks

**DOI:** 10.3389/fmicb.2020.01483

**Published:** 2020-06-30

**Authors:** Déborah Merda, Arnaud Felten, Noémie Vingadassalon, Sarah Denayer, Yacine Titouche, Lucia Decastelli, Bernadette Hickey, Christos Kourtis, Hristo Daskalov, Michel-Yves Mistou, Jacques-Antoine Hennekinne

**Affiliations:** ^1^French Agency for Food, Environmental and Occupational Health & Safety (ANSES), University of Paris-Est, Maisons-Alfort, France; ^2^Scientific Service of FoodBorne Pathogens, Sciensano, Brussels, Belgium; ^3^Laboratory of Analytical Biochemistry and Biotechnology, University of Mouloud Mammeri, Tizi Ouzou, Algeria; ^4^National Reference Laboratory for Coagulase-Positive Including Staphylococcus aureus, Istituto Zooprofilattico Sperimentale del Piemonte, Liguria e Valle d’Aosta, Turin, Italy; ^5^Department of Agriculture, Food and the Marine, Kildare, Ireland; ^6^State General Laboratory, Food Microbiology Laboratory, Nicosia, Cyprus; ^7^National Center of Food Safety, NDRVI, BFSA, Sofia, Bulgaria

**Keywords:** staphylococcal enterotoxins genes, gene detection, variant diversity, bioinformatics tool, genomic

## Abstract

Food contamination by staphylococcal enterotoxins (SEs) is responsible for many food poisoning outbreaks (FPOs) each year, and they represent the third leading cause of FPOs in Europe. SEs constitute a protein family with 27 proteins. However, enzyme immunoassays can only detect directly in food the five classical SEs (SEA-SEE). Thus, molecular characterization methods of strains found in food are now used for FPO investigations. Here, we describe the development and implementation of a genomic analysis tool called NAuRA (Nice automatic Research of alleles) that can detect the presence of 27 SEs genes in just one analysis- and create a database of allelic data and protein variants for harmonizing analyses. This tool uses genome assembly data and the 27 protein sequences of SEs. To include the different divergence levels between SE-coding genes, parameters of coverage and identity were generated from 10,000 simulations and a dataset of 244 assembled genomes from strains responsible for outbreaks in Europe as well as the RefSeq reference database. Based on phylogenetic inference performed using maximum-likelihood on the core genomes of the strains in this collection, we demonstrated that strains responsible for FPOs are distributed throughout the phylogenetic tree. Moreover, 71 toxin profiles were obtained using the NAuRA pipeline and these profiles do not follow the evolutionary history of strains. This study presents a pioneering method to investigate strains isolated from food at the genomic level and to analyze the diversity of all 27 SE-coding genes together.

## Introduction

*Staphylococcus aureus* strains can be responsible for staphylococcal food poisoning by secreting enterotoxins in food matrices. The symptoms caused by the consumption of food contaminated by staphylococcal enterotoxins (SEs) include nausea, vomiting, abdominal cramping, and diarrhea. Symptoms appear quickly after ingestion, between 30 min and 8 h. SEs represent the third leading cause of food poisoning outbreaks (FPOs) in Europe, and they are the second leading FPO cause in France after Salmonella (Kérouanton et al., 2007). Since 2010, the number of FPOs caused by bacterial toxins have significantly increased in France (EFSA and ECDC, 2019). In 2018, bacterial toxins caused 799 FPOs in France and 935 in Europe, of which 114 FPOs were caused by SEs (EFSA and ECDC, 2019).

Currently, 27 SEs have been described (SEA to SEE and SEG to SElX, SElY, SElZ, SEl26, SEl27) and they compose a superfamily of secreted single-chain globular proteins whose molecular weights vary from 19 to 29 kD. After ingestion, SEs have two major toxic activities: (i) a neurotoxic activity that activates the vagus nerve and the emetic center of the brain, triggering vomiting reflexes, (ii) a superantigenic activity leading to the non-specific activation of T lymphocytes, causing a strong fever. Although all SEs have a superantigenic function, not all have emetic functions (Hennekinne et al., 2012). Tests for emetic functions have been performed on 18 SEs in *Suncus murinus* and primates (Omoe et al., 2013; Ono et al., 2017).

SEs are coded by genes of about 700–800 bp, localized on mobile genetic elements (MGEs), such as plasmids, prophages or pathogenicity islands (SaPIs) (Bania et al., 2006; Driebe et al., 2015). Within the same MGE, several SE-coding genes can be found, i.e., the plasmid pF5 harbors the genes coding for SER, SET, SES, and SElJ (Argudín et al., 2010). The genomic island carrying the enterotoxin gene cluster (*egc*) frequently harbors five genes coding for SEG, SEI, SEM, SEN, and SEO. Some variants of this genomic island have the gene coding for SElV but not the genes coding for SEI and SEM (Argudín et al., 2010).

All genes coding for enterotoxins may originate from the *egc*, which is considered as an enterotoxin nursery and can diverge from *egc* by duplication, transposition and mutation (Jarraud et al., 2001; Grumann et al., 2014). Thus, these evolutionary mechanisms have led to several genetic clusters in enterotoxin genes (three reported by Jarraud et al., 2001 and five by Ono et al. (2008), including genes belonging to the *egc* cluster. Furthermore, SEs present different levels of divergence (Ono et al., 2008). For example, SEA and SEE share 81% of homology with regard to their amino-acid sequences, whereas SEB and SEC share 67% of homology (Balaban and Rasooly, 2000).

To detect SE genes, it is necessary to consider the different divergence levels that can best define possible toxigenic profiles and thus help identify SEs implicated in an outbreak. In a FPO investigation, screening for virulence factors is very important. SE genes are currently detected in strains using PCR method developed by the European Reference Laboratory for Coagulase Positive Staphylococci (EURL CPS) and described in Roussel et al. (2015). This method has been used to investigate Italian and Belgian outbreaks (Bianchi et al., 2014; Carfora et al., 2015; Denayer et al., 2017; Monistero et al., 2018). This method can only detect 11 SE genes. Therefore, no information is available on the 16 other genes for FPO investigations. Other PCR methods have been developed to detect 18 of the 27 SE genes (e.g., Zhang et al., 2018). However, the PCR approach has several limitations. First, it does not provide any information on nucleotide variability; second, the amplification of pseudogenes leads to false positives; and finally, mutations in the primer binding sites can produce false negatives.

Genomic approaches to detect virulence factors are under development. Advances in whole genome sequencing (WGS) make it possible to access the complete toxin repertory of a given strain. WGS has been used for several years for monitoring purposes or for outbreak investigations on several pathogens, because this method offers an alternative to other molecular biology methods, such as pulsed-field gel electrophoresis (PFGE) or PCR (Oakeson et al., 2017). Bioinformatics pipelines have been set up by European Reference Centers, e.g., for *Neisseria meningitidis* (Bogaerts et al., 2019).

A specific pipeline called Staphopia was developed for *Staphylococcus* genomic analysis, but no tool for enterotoxin detection has been implemented in it (Petit and Read, 2018). However, for FPO investigations, it is important to identify the enterotoxin-coding genes to establish the link between the SE found in the incriminated food and the strain, and to study other SEs potentially present in food for which immunoassay methods have not been developed. To detect virulence factors in genomic sequences two approaches can be used: (i) mapping reads to reference sequences, e.g., as implemented in ARIBA (Hunt et al., 2017) and (ii) local alignment using BLAST. A benchmark study based on *S. aureus* strains has shown that the choice of method (mapping or local alignment) has no impact on the results (Mason et al., 2018). When genome assemblies are available, the BLAST method is faster than mapping and, with public databases, assembled genomes are more accessible than raw data. Therefore, the objective of this work was to develop a bioinformatics pipeline called NAuRA (Nice Automatic Research of Alleles) to detect genes coding for SEs in *S. aureus* genomes. NAuRA can screen every new genome for its toxin content and toxin allele profile. Moreover, NAuRA can create an allelic database to harmonize protein variant nomenclature of SEs, and to facilitate information sharing between outbreak cases. NAuRA was implemented to study enterotoxin-coding gene families in *S. aureus* strains responsible for FPOs in Europe between 2005 and 2017.

## Materials and Methods

### Bioinformatics Development

**FIGURE 1:**
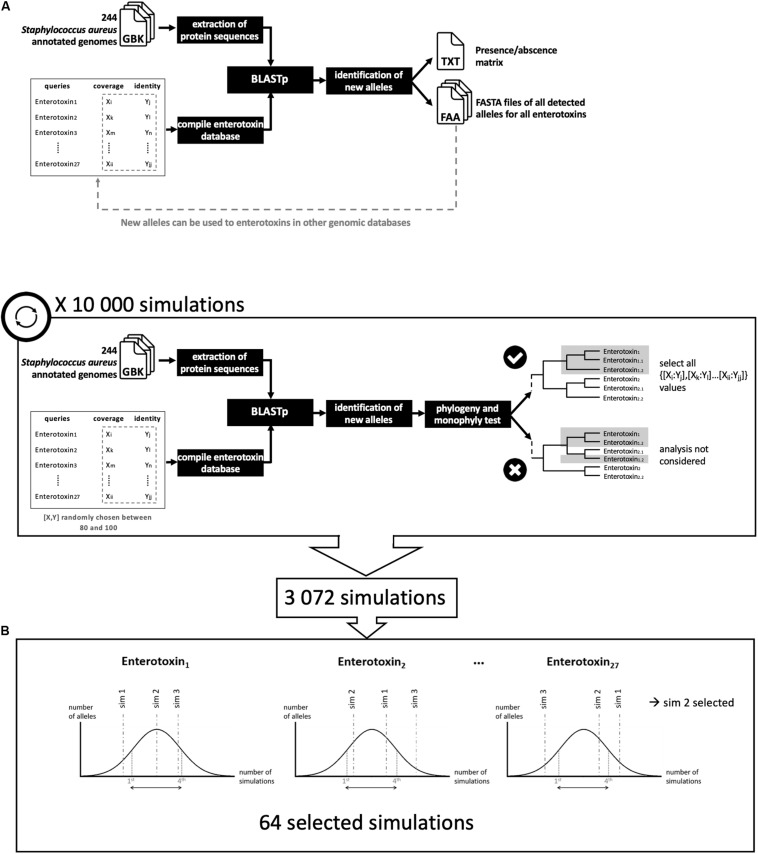
Analytical schematic of staphylococcal enterotoxin (SE) gene detection. **(A)** NAuRA (Nice Automatic Research of Alleles). **(B)** Determination of the best parameters to filter the BLAST results.

An in-house workflow NAuRA was developed in Python to automate and harmonize the screening for genes of interest in a collection of genomes (Figure 1). Based on BLAST, NAuRA detects the presence of genes/proteins from a user-defined query list in the user-defined collection of genomes and provides an allele identifier when a matching gene/protein is found. Every allele-type sequence having a new identifier is added to the database of queries. Furthermore, the workflow offers the possibility to perform a quick clustering analysis using a neighbor-joining approach based on allele sequences. NAuRA is compatible with both nucleic and protein sequences and is available on github^1^.

From genomes in GenBank format and a list of query genes of interest in FASTA format, NAuRA converts GenBank files to FASTA nucleic acid (fna) or FASTA amino acid (faa) files where each sequence represents an open reading frame (ORF) previously predicted by Prokka. Then, sequences of interest are aligned to the chosen genomes using BLAST + (Camacho et al., 2009) and the results are filtered with two thresholds: (1) the minimum percentage of similarity and (2) the minimum percentage of coverage. If a gene is considered as present, i.e., if the two percentages of similarity and coverage are greater than or equal to the two thresholds, the ORF is extracted and compared to all known alleles using BLAST. If a new allele is detected, i.e., if a mutation in an amino acid is detected, it is added to the corresponding query FASTA file and a new identifier is assigned. After the alignment step, NAuRA outputs for each genome a gene presence/absence matrix in which the allele identifier is specified. NAuRA offers the option to align all alleles of each query using Clustal Omega (Sievers et al., 2011) to construct a phylogenetic tree using the neighbor-joining method implemented in Phylip (Felsenstein, 2013). Node support values are calculated using the bootstrap method. The consensus tree is obtained using the sumtree.py script from the DendroPy Library (Sukumaran and Holder, 2010).

To analyze a gene family with an unknown level of divergence, a second Python script was developed, NAuRA_BPF (Figure 1B). To optimize the setting of threshold values, BLAST analysis was performed with a series of coverage and similarity thresholds randomly chosen in uniform distribution. The best analyses corresponding to specific threshold values were selected using a monophyly test, which assumes that all alleles belonging to a given gene family form a monophyletic branch on a phylogenetic tree.

### Strain Collection and DNA Extraction

A collection of 143 genomes belonging to *S. aureus* and the cause of FPO in Europe from 2005 to 2017 were collected by the EURL CPS and conserved at −80°C. This collection represented the known diversity of strains isolated from food in Europe and was typed using PFGE; some were reference strains (Supplementary Table S1). For DNA extraction, strains were regenerated on Milk Plate Count Agar for 24 h at 37°C. To purify the culture, one colony was placed on brain heart infusion (BHI) agar for 16–20 h at 37°C. DNA extraction was performed using the Wizard^®^ Genomic DNA Purification kit (Promega) from the colonies on BHI agar. A pre-lysis step was added with a treatment of 105 μl of lysozyme and 15 μl of lysostaphin for 30 min at 37°C. DNA quality was evaluated using a NanoDrop spectrophotometer and the concentration was quantified using a Qubit fluorometer. DNA integrity was evaluated by electrophoresis on 0.8% agarose gel.

### Genome Sequencing, Assembly, and Annotation

Genomes were sequenced using Illumina NextSeq system (ICM institute, Paris, France) and the Nextera XT DNA Library Prep Kit was used to construct libraries. The sequencing data were presented as reads of 2^∗^150 bp. Before assembly, reads were normalized using BBnorm^2^ to reduce coverage to 100x and trimmed using Trimmomatic (Bolger et al., 2014) to remove bases having a Phred score of less than 30 and to remove reads smaller than 50 bp. Assembly was performed in three steps. First, the *de novo* assembly was processed using Spades with default parameters. Then, the nearest complete public genome of *S. aureus* was found using Mash (Ondov et al., 2016) and used to perform the scaffolding step in MeDuSa (Bosi et al., 2015). Finally, a gap-closing step was processed with GMcloser (Kosugi et al., 2015). Genome assemblies were evaluated using Quast (Gurevich et al., 2013) and scaffolds smaller than 200 bp were removed. Genome annotation was performed using Prokka (Seemann, 2014). Raw reads and assembly data are available in the European Nucleotide Archive (ENA) under the study number PRJEB36867.

Publicly available genomes were also added to the dataset: 101 assembly genomes available from the RefSeq database in NCBI. They were re-annotated using Prokka to ensure a homogeneous dataset. Multilocus sequence typing (MLST) was performed on each sample, the sequence type (ST) was assigned using the MLST program^3^, and the database PubMLST^4^ and the scheme of seven housekeeping genes (*arcC*, *aroE*, *glpF*, *gmk*, *pta*, *tpi*, and *yqiL*) developed for *S. aureus* (Enright et al., 2000).

Thus, our total collection included 101 genomes from public databases and 143 genomes sequenced in-house. For strains sequenced in-house, they were isolated in Europe; 103 were potentially responsible for an FPO, 14 were reference strains, 15 were isolated from self-testing and 10 had been used in a previous study (Titouche et al., 2019). The quality of sequencing, assembly and annotation was robust: the coverage of reads was on average 329x (Supplementary Table S1). Regarding the assembly, the expected size of *S. aureus* genomes was found (on average 2.71 Mb) and the number of contigs varied between 1 and 69. The dataset annotation was harmonized using the same software (i.e., Prokka), and the number of annotated genes varied between 2248 and 2751.

### Screening for Genes of Interest

We screened for the previously described 26 enterotoxins- and TSST1-coding genes using protein sequences as queries on the 143 *S. aureus* genomes isolated from food matrices and on the 101 publicly available genomes (Supplementary Table S1). The accession numbers of these genes are given in Supplementary Table S1. Reviewed protein sequences available in public databases were chosen. For the other SEs for which no reviewed sequences were available, their genomic localization, and for two new SEls, *sel26*, and *sel27*, the protein sequences were taken from the literature (Zhang et al., 2018). Because these genes are genetically close, parameter adjustment was performed to discriminate all alleles of each gene using the Python script NAuRA_BPF. Coverage and similarity parameters were tested for values between 80 and 100 to perform 10,000 simulations. Among these simulations, monophyletic groups were found for each gene in 3072 simulations, the other 6928 analyses were removed. The best analysis was selected to maximize allelic diversity for each gene. For each gene, a distribution of the number of different alleles was established using the 3072 selected simulations. A random choice among analyses providing a number of alleles between the first and fourth quartile of the distribution was performed. This procedure conserves the diversity of alleles and reduces the stringency of the selection parameters. Nucleotide sequences were extracted from GenBank files, and using BLAST best thresholds result obtained by NAuRA for the best analysis.

### Determination of Thresholds for Detecting Staphylococcal Enterotoxins

NAuRA is a Python pipeline to detect sequences of interest in genomes with a BLAST approach. It was developed to mainly detect SE genes. With the different divergence levels, simulations were performed to optimize filter parameters of identity and coverage. Accordingly, among the 10,000 simulations, 64 were conserved after the monophyly test (Figure 1). The values of the parameters used for the 64 simulations covered the whole uniform distribution between 80 and 100 for identity and coverage parameters, except for the four following genes: *sea*, *see*, *sep*, and *selv*. This means that the 64 selected simulations were based mainly on these four genes; for this reason, for the final analysis, the lowest threshold to maintain the allele monophyly was 80% of identity and coverage for the 23 SE genes, 90% of identity for *selv* and 84% for identity for *sea*, *sep*, and *see*. These values confirmed the low divergence levels between *sem* and *selv* and between *sea*, *sep*, and *see.* On the Supplementary Figure S1, three phylogenies based on protein variants were presented and corresponded to three situations: (i) released parameters, (ii) stringent parameters, and (iii) selected parameters. The released parameters were percentage of identity inferior to 84% for all genes (80% of identity). The stringent parameters were percentage of identity superior to 84% for all genes (between 95 and 100%). And the selected parameters were parameters described before. With released parameters, no differentiation between *sea*, *see*, and *sep* variants was observed (Supplementary Figure S1A). With stringent parameters, some genes, such as *seo* and *selu*, exhibit few variants, with the consequence to increase the false-negative results (Supplementary Figure S1B). The phylogeny based on variants obtained with selected parameters described below allows to maximize diversity while maintaining monophyly (Supplementary Figure S1C).

### Comparisons With PCR Method and Toxin Detection

Profiles obtained by NAuRA were compared to PCR-detected genes according to the methods described in Roussel et al. (2015), This method allows the detection of 11 SE-genes (*sea*, *seb*, *sec*, *sed*, *see*, *seg*, *seh*, *sei*, *selj*, *sep*, *ser*). PCR results were available for each strain of the genome collection sequenced for this study., Toxin detection in food was performed according the standard ISO 19020 for the “classical” toxins (SEA to SEE), and results were available for 54/143 strains. These results were also compared to profiles obtained by NAuRA.

### Core Genome Definition and Phylogenetic Analyses

The core genome of the 244 strains, which is defined by all genes shared by all genomes in only one copy, was determined using Roary (Page et al., 2015) applied on the GFF3 files obtained in Prokka. Based on the pBLASTn approach, Roary extracts the pan-genome and aligns all its ORF sequences using MAFFT (Katoh et al., 2002). To remove the misaligned regions, the Gblock program (Castresana, 2000) was used on this alignment. To obtain a robust phylogenetic tree with the maximum-likelihood method, iQtree (Chernomor et al., 2016) was run. The node support values were calculated using the bootstrap method. Bootstrap values were calculated with 1000 replicates and a bootstrap convergence test was carried out to check if this number of replicates was sufficient. The representation of this tree was obtained using the iTOL web viewer. The enterotoxin presence/absence coding genes and FPO information was added on the phylogenetic tree.

### Cophenetic and Robinson Foulds Metric

Comparison of phylogenetic tree based on core genome and dendrogram based on enterotoxin profiles was performed using the cophenetic index. This index could vary between −1 for perfect negative correlation and 1 for perfect positive correlation. A cophenetic index of 0 indicates an absence of correlation between topologies. Then, this index allows evaluating the correlation between the core genome and the toxigenic profile. The Robinson Foulds (RF) metric was also calculated, and this metric indicates the number of distances between topologies. The cophenetic index and the RF metric were calculated with the R packages *phangorn* and *dendextend* (Cardona et al., 2013).

## Results

### Description and Justification of the Dataset

Based on the core genome, the dataset was representative of the known diversity of *S. aureus* species. Furthermore, MLST analysis was performed on all sequences and 62 different STs were found in the collection, although it was not possible to determine the ST for 22 strains. The clonal complex was estimated for these strains. Among the 62 different STs, the 10 most frequent STs found in 40,000 genomes of *S. aureus* (Petit and Read, 2018) were the following: ST22, ST8, ST5, ST239, ST398, ST30, ST45, ST15, ST36, and ST105. However, some STs frequent in food, which were not detected in Petit and Read (2018) study were also identified, e.g., ST1 and ST389.

**FIGURE 2:**
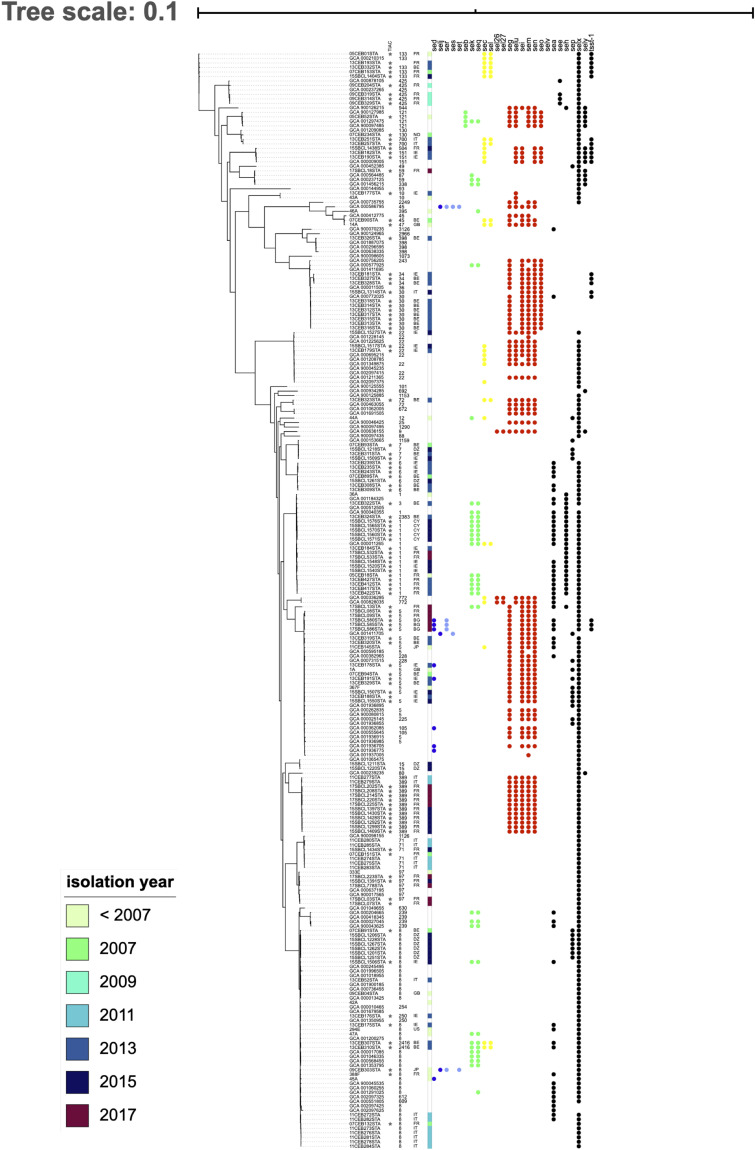
Phylogeny of *Staphylococcus aureus* and distribution of staphylococcal enterotoxin (SE) genes. The phylogenetic inference based on the core genome was obtained using Roary on 244 of *S. aureus* genomes. The stars indicate strains involved in food poisoning outbreaks (FPOs). Each filled circle indicates the presence of SE genes; each color indicates SE genes linked on the same mobile genetic element (MGE). SE genes located on plasmid pIB485 are shown in blue; SE genes located on the same pathogenicity island in yellow or green; SE genes located on the *egc* cluster in red and all other genes not located on an MGE in black.

Strains involved in FPOs formed a paraphyletic group (starred in Figure 2). In the maximum likelihood tree, strains responsible for FPOs were distributed throughout the tree and were mixed with strains isolated from humans or environment (Figure 2). For example, strains occurring in France in 2017 (17SBCL13STA, 17SBCL08STA, 17SBCL09STA) constituted a monophyletic group with genomes from the RefSeq database and also with strains responsible for FPOs in Bulgaria in 2017 (17SBCL580, 17SBCL585, and 17SBLC586) and in Belgium in 2013 (13SBCL319STA and 13SBCL320). This pattern suggests that strains are not structured according the country of isolation or year of isolation. For instance, strains isolated in 1997, 2011, 2013, and 2015 belonging to ST8, are monophyletic with genomes from the RefSeq database.

### Toxin Profiles of Strains

Toxin profiles obtained by NAuRA are highly in accordance at 93.7% with profiles obtained by PCR approach. Indeed, only nine different profiles out of 143 were observed between the two methods. Of the nine profiles, eight corresponded to a gene detected by PCR but not by NAuRA, four of them implicated *seg* gene, two of them implicated *sed* gene, one implicated *sec* gene, and the last implicated two genes: *sea* and *seh*. The 9th different profile corresponded to no detection of *sep* gene by PCR. Concerning the comparison of NAuRA results and toxin detection in food, eight results were discordant from 54 available results. Then, 85.2% of results were in accordance. Among these eight discordances, six corresponded to no toxin detection whereas *se* gene was detected by NAuRA and PCR methods, and the other two corresponded to SEC detection, whereas *sec* gene was not detected in the strain, suggesting presence of other strains in the food.

The gene distribution did not follow the phylogeny (Figure 2) as expected for genes found on mobile genetic elements (MGE); subject to horizontal gene transfer. The cophenetic index comparing the phylogeny based on the core genome and the dendrogram based on toxin profiles was 0.25. The closer this index is to 0, the more the two compared trees are statistically different. The symmetric difference, or the Robinson Foulds metric, was 436, suggesting that 436 branches differed between the phylogeny and the dendrogram. This index does not take branch length into account, and only compares the topologies. The higher the number of this metric, the more the topologies differ.

The *egc* cluster (in red in Figure 2) was present in several groups of strains: 36% of strains had the *egc* cluster and it is absent in the two monophyletic groups, suggesting the loss of this cluster during the evolutionary history of *S. aureus*. Regarding genes located on plasmids, several toxin profiles were found within the same strain group. For example, the strains 11CEB284STA and 47A clustered together, but only one of them had the *sed*, *selj*, and *ser* genes and the other had *seb*, *sek*, and *seq*.

No difference in toxin profiles was observed between strains responsible for FPO and the other strains. Strains from the RefSeq database had the same toxin profile as FPO strains. For example, 15SBCL1507STA and GCA_00262835 both showed the *egc* cluster and the SElX-coding gene; the 13CEB176STA and GCA_001350955 strains had the genes coding for SEB, SEK, and SEQ. As no toxin profile can be shared by environmental strains and FPO, this could suggest that food contamination occurs via environmental strains.

**FIGURE 3:**
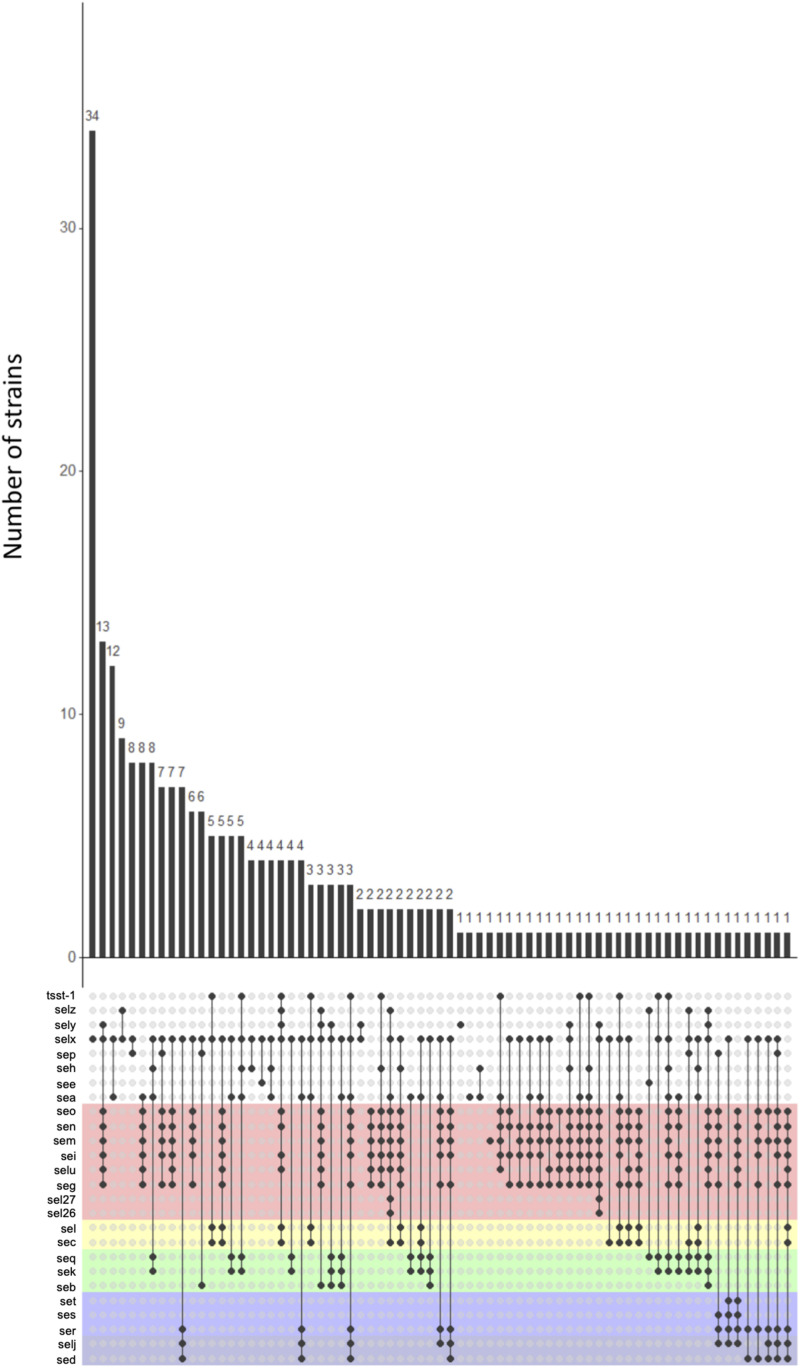
Association pattern of staphylococcal enterotoxin (SE) genes. The UpSet plot shows all combinations of SE genes found in 244 genomes; the histogram shows the number of strains in which the combination was found. Each color background represents the SE genes linked on a given mobile genetic element (MGE). SE genes linked to no other and distributed in the genome are shown against a white background.

A large number of toxin profiles were found (71 profiles, of which 34 were found only once) (Figure 3). The *selx* gene was found in the majority of profiles (53/71), as well as the *egc* cluster (39/71). Some genes were never found together. Indeed, the *ser*, *sed*, and *selj* genes localized on plasmid were never found with the *seq*, *sek*, and *seb* genes localized on SaPI. This non-association suggests that strains cannot carry both MGEs simultaneously. Moreover, the *sea*, *see*, and *sep* genes which are genetically close, were never found together within the same strain.

The number of enterotoxins for each strain varied between 0 and 11 (Figure 2 and Supplementary Table S2). Overall, 15.9% (39/244) of strains had only one enterotoxin corresponding mainly to SElX, 21% strains had at least five enterotoxins corresponding to the *egc* cluster and the SElX coding gene. Gene frequency in the collection varied among the different SE-coding genes. For example, 86% (210/244) of strains had the SElX-coding gene and 30% (74/244) of strains carried the gene coding for SEA. Concerning genes of *egc* cluster, 34% carried the gene coding for SEG (83/244), 34.8% (85/244) carried the gene coding for SEM, 36.4% (89/244) carried the gene coding for SEN and 36.4% (89/244) carried the gene coding for SEO. But genes coding for SES and SET were only present in three and two strains respectively and the gene coding for SElV was absent in the collection.

### Enterotoxin Sequence Analyses

**FIGURE 4:**
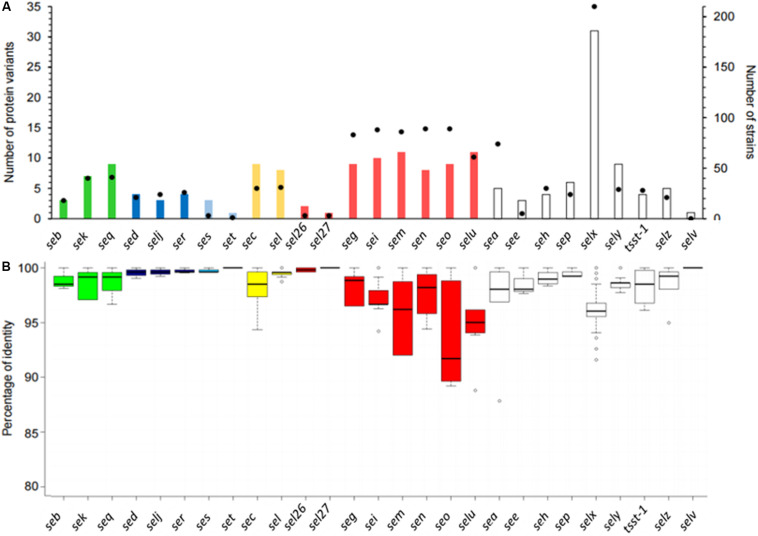
Representation of stapylococcal enterotoxin (SE) protein diversity. **(A)** Histogram showing the number of protein variants (left axis) found in the 244 genomes according to the SE gene. The filled black circles (right axis) correspond to the number of strains in which the SE gene was found. **(B)** Boxplots show the distribution of the percentage of identity between the protein variants and the reference sequence. Each color represents the SE genes linked on the same mobile genetic element (MGE). SE genes located directly in the bacterial genome are shown in white.

The number of variants differed according to the SEs (Figure 4). The number of protein variants varied between 1 and 11, except for the SElX-coding gene for which 31 protein variants were found (Figure 4). For the *egc* cluster comprising *seg*, *sei*, *sem*, *sen*, and *seo*, the same magnitude of protein. A similar number of variants in SED/SElJ pair localized on pIB485, and the SEC/SEL pair localized on SaPIbov was observed. It is also possible for genes localized on the same plasmids to have a different number of variants. For example, one protein variant was found in the dataset for SET, whereas four variants were found for the other toxins localized on the same plasmid (SElJ, SER and SES).

In all, 16 SE genes (*sea*, *seb*, *see*, *seg*, *seh*, *sei*, *selj*, *sem*, *sen*, *seo*, *sep*, *ser*, *set*, *selz*, *sel26*, and *sel27*) had one dominant protein variant in the dataset (at least 50% of strains), whereas no dominant protein variant was observed for the 11 other SE genes (*sec*, *sed*, *sek*, *see*, *seq*, *ses*, *selu*, *selx*, *sely*). Therefore, the predominance of some alleles may facilitate the choice of reference sequences for detection methods in food.

## Discussion

SEs are responsible for FPOs and their presence in food is regulated by European legislation (Ostyn et al., 2010). If, for example, SEs are found in a 25 g sample of cheese, this contaminated food cannot be marketed, potentially representing high economic losses for the food company. Therefore, it is important to set up a robust and sensitive methods to detect enterotoxins. Currently, only five enterotoxins are directly detectable in food. To help outbreak investigation and establish a link between toxins and strains, molecular biology methods have been developed to detect SE-coding genes in strains, mainly using PCR. These methods are costly in terms of development and laboratory time. On the other hand, in this genomics era, both nucleotide and protein sequences are available for a specific strain. In contrast to toxin detection, molecular biology methods and genomic require strain cultivation and DNA extraction before analysis. Genomic and molecular biology methods were strongly in accordance, and differences were observed in particular for *seg* gene. This cluster is submitted to recombination events, and *se* gene fragments were found, suggesting pseudogeneization, false positive results in PCR. More differences were observed between gene content and toxin detection in food. This could be explained by the fact that not all the required conditions for toxin secretion were present. Indeed, SEs would not all secrete at the same time during the growth (Derzelle et al., 2009).

NAuRA is the first pipeline that can detect all 27 currently described SE genes and the simulation parameter adjustment can establish specific parameters for genetically close genes. The Staphopia pipeline, specifically developed for *S. aureus*, detects only nine SE genes available in the VFDB 2016 database (Chen et al., 2015) (*sea*, *seb*, *sec*, *sed*, *see*, *seh*, *sek*, *seq*, *tsst-1*), because this pipeline was mainly developed to analyze antibiotic resistance (Petit and Read, 2018). SeqSphere software was mainly developed to analyze core-genome MLSTs and can be also used to detect 18 enterotoxins (Strauß et al., 2017). However, the task template for *S. aureus* and based on sequences of a microarray system does not distinguish sea from sep genes, thus considered as the same gene.

It is very important to detect all SE-coding genes and to describe all existing SEs to assess the prevalence of toxigenic strains. An increase in the number of toxins detected may mean an increase in the percentage of toxigenic strains (Le Loir et al., 2003; Bania et al., 2006; Aydin et al., 2011). For example, the number of toxigenic strains identified in our study (97.5% of strains) is higher than that reported in precedent studies: although based on the genes coding for the five classical SE genes (SEA to SEE), the toxigenic strain frequency was around 60% in several countries, such as Turkey, Portugal and Italy (62.6, 60.1, and 59.8%, respectively). In Iran and Louisiana (United States), the detection of nine SEs showed that 77.6 and 84.9%, respectively, of strains were toxigenic in these regions (Pu et al., 2010; Mashouf et al., 2015). In another study, the SElX-coding gene is present in 95% of strains, and it was localized in the core genome (Wilson et al., 2011). Thus, screening for SElX-coding genes in the dataset can reveal a high percentage of toxigenic strains. Currently, only the five classical SEs are detected directly in food for FPO investigations, and the toxigenic power of non-detected strains is not known.

Among the five genes coding for the classical SEs, the genes encoding the SEA and SEC toxins were the most frequent. This distribution is similar to that in a Jordanian study where these genes were detected by PCR (Naffa et al., 2006). However, the prevalence of *sea* is higher than *sec* in our study. The SEA-coding gene may indicate a human origin for the strain, whereas the SEC-coding gene may indicate a bovine origin for the strain. Therefore, strains responsible for FPOs in Europe likely have a human origin, as expected for FPOs (Le Loir et al., 2003; Ortega et al., 2010), because food contamination by *S. aureus* can often be retraced to hygiene problems.

For FPO investigations, it is important to detect all SE-coding genes in the *egc* cluster so as to avoid underestimating its prevalence. There are several profiles of *egc* in strains responsible for FPOs. The obtained profiles for the *egc* cluster corresponded to the profiles reported in Song et al. (2016), with only four additional profiles being found just once. This diversity confirms that *seg* and *sei* genes are not always linked, as shown in Adwan et al. (2013). Our frequencies of *egc1* and *egc2*, as defined by Song et al. (2016), were similar to those obtained in their study with 28 and 55%, respectively. These values correspond to the frequencies obtained for strains isolated from food and corroborate our data, because a large part of strains were also isolated from food. Likewise, the *egc* cluster profile shows higher diversity in strains isolated from food than other environments (Song et al., 2016). NAuRA provides access to the complete nucleic diversity of a specific dataset. This can be useful for the development of new, more efficient PCR methods, for example, by designing primers preferentially in the more conserved region of allele sequences. The study of nucleic diversity can also help to choose the reference protein sequence to use for immunoassays methods, for example. In the UniProt database, only nine SEs are annotated and can serve as a standard for method development (The UniProt Consortium, 2018). Moreover, to harmonize analyses, NAuRA can create a database of newly identified variants to include them in subsequent analyses.

The complete *egc2* cluster is the most frequent *egc* variant and is distributed throughout the phylogenetic tree based on the core genome, as revealed in trees based on MLVA and PFGE. In contrast, the *egc1* cluster formed a monophyletic group on the core-genome tree, but was scattered in Song et al. (2016) study, based on MLVA and PFGE on SEs. Several evolutionary scenarios may be responsible for this distribution pattern. The first scenario involves an *egc* cluster without the *selu* gene and several acquisitions at different phylogenetic levels. The second scenario involves a complete ancestral *egc* cluster and subsequent loss of genes during evolution. The latter scenario is the most parsimonious hypothesis, because it involves only one evolutionary event. This scenario also provides support for the hypothesis according to which the *egc* cluster is an SE nursery (Jarraud et al., 2001; Grumann et al., 2014) and a highly dynamic region subject to duplication and transposition events.

The number of toxin profiles obtained was lower than the number reported in China (71 versus 120, respectively) (Chao et al., 2015). This difference can be explained by the fact that strain diversity in China is higher than the diversity in our genome collection or by PCR limitations, as PCR detects only a small gene portion whereas NAuRA detection is performed on the predicted proteome by structural annotation However, the presence of a gene in a genome does not necessarily imply its expression. In the case of FPOs, bacterial strains make food unfit for consumption by directly secreting the toxin into the food (Fisher et al., 2018). Therefore, linking a strain to a toxin requires transcriptomic methods to verify enterotoxin gene expression.

Finally, NAuRA may be sensitive to sequence assembly biases due to a large number of contigs (fragmentated assembly). Thus, poor-quality assemblies can reduce the number of detected genes if they are located at the ends of contigs. NAuRA is also limited to detecting only genes annotated in Prokka, ignoring any non-annotated genes actually present in the strain. This assembly anomaly can occur in repeat sequence regions such as insertion sequences (Is) or VNTR regions (Hawkey et al., 2015). In the case of poor assembly quality or Is/VNTR-coding gene searches, it would be more reasonable to use a mapping approach such as GeneFinder (Mason et al., 2018).

## Conclusion

In conclusion, SE gene detection using bioinformatics analyses highlighted SE gene prevalence in European strains of *S. aureus*. These results can be used to improve fast detection methods, such as RT-PCR, microarray analyses or digital PCR, for SE genes. They can be used to determine the new target for the development of molecular detection methods, and improve outbreak investigation in Europe.

## Data Availability Statement

The datasets generated for this study can be found in the European Nucleotide Archive (ENA) under accession number PRJEB36867 (https://www.ebi.ac.uk/ena/data/view/PRJEB36867).

## Author Contributions

DM and AF designed the project, developed the pipeline, analyzed the data, and wrote the manuscript. NV participated in strain choice and performed the DNA extraction and preparation for sequencing. SD, YT, LD, BH, CK, and HD provided strains for sequencing. M-YM helped to draft the manuscript and participated in the coordination of the study. J-AH designed the study, helped to draft the manuscript, and coordinated the study. All authors contributed to the article and approved the submitted version.

## Conflict of Interest

The authors declare that the research was conducted in the absence of any commercial or financial relationships that could be construed as a potential conflict of interest.

## Footnotes

1 https://github.com/afelten-Anses/NAuRA

2 https://jgi.doe.gov/data-and-tools/bbtools/

3 https://github.com/tseemann/mlst

4 https://pubmlst.org/saureus/
