# Supplementary material for: NAuRA: Genomic Tool to Identify Staphylococcal Enterotoxins in Staphylococcus aureus Strains Responsible for FoodBorne Outbreaks
Source: Front Microbiol. 2020 Jun 30;11:1483. doi: 10.3389/fmicb.2020.01483 (PMC7344154; doi:10.3389/fmicb.2020.01483)
Supplement: Supplementary file 1 [file Table_1.docx]

Supplementary Material

Table I: Epidemiological data of 143 sequenced strains for this study

| Strain | Year of isolation | Country | Context of isolation | Source | Sequence Type (ST) or Clonal Complex (CC) | Contig number | Size (Mb) | Gene number | Coverage (X) | Accession no. |
| --- | --- | --- | --- | --- | --- | --- | --- | --- | --- | --- |
| 05CEB01STA | 2005 | France | FPO | dairy product | 133 | 30 | 2,8127 | 2326 | 243 | ERS4338873 |
| 05CEB18STA | 2005 | France | FPO | meat | 1 | 3 | 2,7496 | 2547 | 250 | ERS4338874 |
| 05CEB52STA | 2005 | NA | FPO | human | 121 | 4 | 2,8009 | 2290 | 244 | ERS4338875 |
| 07CEB132STA | 2007 | France | FPO | dairy product | 8 | 9 | 2,7644 | 2596 | 167 | ERS4338876 |
| 07CEB151STA | 2007 | France | FPO | dairy product | CC97 | 8 | 2,7537 | 2412 | 271 | ERS4338877 |
| 07CEB153STA | 2007 | France | FPO | dairy product | 133 | 13 | 2,8019 | 2325 | 466 | ERS4338878 |
| 07CEB234STA | 2007 | Norway | FPO | dairy product | 130 | 2 | 2,7619 | 2280 | 211 | ERS4338879 |
| 07CEB89STA | 2007 | Belgium | FPO | ready-to-eat food | 6 | 7 | 2,7195 | 2379 | 386 | ERS4338880 |
| 07CEB90STA | 2007 | Belgium | FPO | ready-to-eat food | 45 | 1 | 2,6623 | 2248 | 199 | ERS4338881 |
| 07CEB91STA | 2007 | Belgium | FPO | ready-to-eat food | 8 | 3 | 2,7778 | 2637 | 394 | ERS4338882 |
| 07CEB93STA | 2007 | Belgium | FPO | ready-to-eat food | 7 | 8 | 2,7176 | 2360 | 147 | ERS4338883 |
| 07CEB94STA | 2007 | Belgium | FPO | ready-to-eat food | 5 | 9 | 2,8277 | 2497 | 226 | ERS4338884 |
| 09CEB04STA / NCTC8325 | 1943 | United Kingdom | reference strain | human | 8 | 2 | 2,7980 | 2623 | 452 | ERS4338885 |
| 09CEB204STA | 2009 | France | FPO | dairy product | 425 | 38 | 2,7120 | 2368 | 513 | ERS4338886 |
| 09CEB303STA | 1997 | Japon | FPO | NA | 8 | 10 | 2,7824 | 2563 | 485 | ERS4338887 |
| 09CEB314STA | 2009 | France | FPO | dairy product | 425 | 2 | 2,7647 | 2390 | 275 | ERS4338888 |
| 09CEB319STA | 2009 | France | FPO | dairy product | 425 | 3 | 2,7645 | 2391 | 256 | ERS4338889 |
| 09CEB329STA | 2009 | France | FPO | dairy product | 425 | 2 | 2,7647 | 2388 | 255 | ERS4338890 |
| 11CEB145STA / Mu50 | 1997 | Japan | reference strain | human | 5 | 7 | 2,8593 | 2677 | 417 | ERS4338891 |
| 11CEB272STA | 2011 | Italy | self-testing | dairy product | 8 | 1 | 2,7802 | 2589 | 370 | ERS4338892 |
| 11CEB273STA | 2011 | Italy | self-testing | environment | 8 | 1 | 2,7655 | 2598 | 501 | ERS4338893 |
| 11CEB274STA | 2011 | Italy | self-testing | dairy product | 71 | 11 | 2,6423 | 2361 | 289 | ERS4338894 |
| 11CEB275STA | 2011 | Italy | self-testing | dairy product | 71 | 10 | 2,7432 | 2402 | 280 | ERS4338895 |
| 11CEB276STA | 2011 | Italy | self-testing | meat | 8 | 1 | 2,8185 | 2594 | 262 | ERS4338896 |
| 11CEB277STA | 2011 | Italy | self-testing | dairy product | 389 | 7 | 2,7970 | 2491 | 101 | ERS4338897 |
| 11CEB278STA | 2011 | Italy | self-testing | environment | 8 | 2 | 2,7655 | 2598 | 327 | ERS4338898 |
| 11CEB279STA | 2011 | Italy | self-testing | environment | 389 | 6 | 2,7007 | 2498 | 270 | ERS4338899 |
| 11CEB280STA | 2011 | Italy | self-testing | environment | 71 | 6 | 2,7065 | 2410 | 369 | ERS4338900 |
| 11CEB281STA | 2011 | Italy | self-testing | environment | 8 | 1 | 2,766 | 2596 | 305 | ERS4338901 |
| 11CEB282STA | 2011 | Italy | self-testing | environment | 8 | 1 | 2,7892 | 2589 | 371 | ERS4338902 |
| 11CEB283STA | 2011 | Italy | self-testing | environment | 71 | 12 | 2,6905 | 2408 | 171 | ERS4338903 |
| 11CEB284STA | 2011 | Italy | self-testing | environment | 8 | 1 | 2,8285 | 2597 | 279 | ERS4338904 |
| 11CEB285STA | 2011 | Italy | self-testing | environment | 71 | 6 | 2,7096 | 2402 | 307 | ERS4338905 |
| 13CEB175STA | 2013 | Ireland | FPO | NA | 8 | 2 | 2,7954 | 2696 | 311 | ERS4338906 |
| 13CEB176STA | 2013 | Ireland | FPO | NA | 250 | 34 | 2,8716 | 2654 | 291 | ERS4338907 |
| 13CEB177STA | 2013 | Ireland | FPO | NA | 10 | 15 | 2,7925 | 2248 | 329 | ERS4338908 |
| 13CEB178STA | 2013 | Ireland | FPO | NA | 5 | 3 | 2,7586 | 2485 | 251 | ERS4338909 |
| 13CEB179STA | 2013 | Ireland | FPO | NA | 22 | 1 | 2,7970 | 2648 | 337 | ERS4338910 |
| 13CEB181STA | 2013 | Ireland | FPO | ready-to-eat food | 34 | 2 | 2,8560 | 2619 | 174 | ERS4338911 |
| 13CEB182STA | 2013 | Ireland | FPO | dairy product | 151 | 1 | 2,7047 | 2588 | 391 | ERS4338912 |
| 13CEB184STA | 2013 | Ireland | FPO | dairy product | 1 | 3 | 2,6814 | 2464 | 365 | ERS4338913 |
| 13CEB188STA | 2013 | Ireland | FPO | dairy product | CC5 | 4 | 2,7857 | 2514 | 469 | ERS4338914 |
| 13CEB190STA | 2013 | Ireland | FPO | dairy product | 151 | 2 | 2,7120 | 2634 | 216 | ERS4338915 |
| 13CEB191STA | 2013 | Ireland | FPO | dairy product | 5 | 12 | 2,8347 | 2579 | 400 | ERS4338916 |
| 13CEB193STA | 2013 | France | FPO | dairy product | 133 | 41 | 2,7891 | 2317 | 719 | ERS4338917 |
| 13CEB235STA | 2013 | Ireland | FPO | ready-to-eat food | 6 | 5 | 2,6995 | 2393 | 335 | ERS4338918 |
| 13CEB239STA | 2013 | Ireland | FPO | ready-to-eat food | 6 | 5 | 2,7003 | 2390 | 341 | ERS4338919 |
| 13CEB243STA | 2013 | Ireland | FPO | ready-to-eat food | 6 | 5 | 2,7001 | 2393 | 206 | ERS4338920 |
| 13CEB251STA | 2013 | Italy | FPO | dairy product | 700 | 3 | 2,8163 | 2306 | 293 | ERS4338921 |
| 13CEB257STA | 2013 | Italy | FPO | dairy product | 700 | 6 | 2,8157 | 2299 | 221 | ERS4338922 |
| 13CEB307STA | 2013 | Belgium | FPO | ready-to-eat food | 2416 | 3 | 2,8025 | 2666 | 347 | ERS4338923 |
| 13CEB308STA | 2013 | Belgium | FPO | meat | 6 | 5 | 2,7679 | 2437 | 182 | ERS4338924 |
| 13CEB309STA | 2013 | Belgium | FPO | meat | 6 | 11 | 2,7699 | 2436 | 168 | ERS4338925 |
| 13CEB310STA | 2013 | Belgium | FPO | meat | 2416 | 6 | 2,7927 | 2666 | 296 | ERS4338926 |
| 13CEB311STA | 2013 | Belgium | FPO | ready-to-eat food | 7 | 5 | 2,7798 | 2568 | 217 | ERS4338927 |
| 13CEB312STA | 2013 | Belgium | FPO | ready-to-eat food | 30 | 22 | 2,8043 | 2643 | 367 | ERS4338928 |
| 13CEB313STA | 2013 | Belgium | FPO | human | 30 | 14 | 2,8001 | 2643 | 443 | ERS4338929 |
| 13CEB314STA | 2013 | Belgium | FPO | human | 30 | 6 | 2,8026 | 2607 | 136 | ERS4338930 |
| 13CEB315STA | 2013 | Belgium | FPO | human | 30 | 8 | 2,8558 | 2606 | 224 | ERS4338931 |
| 13CEB316STA | 2013 | Belgium | FPO | human | 30 | 6 | 2,8003 | 2606 | 188 | ERS4338932 |
| 13CEB317STA | 2013 | Belgium | FPO | human | 30 | 17 | 2,8009 | 2640 | 438 | ERS4338933 |
| 13CEB318STA | 2013 | Belgium | FPO | human | 30 | 15 | 2,7983 | 2636 | 516 | ERS4338934 |
| 13CEB319STA | 2013 | Belgium | FPO | ready-to-eat food | 5 | 2 | 2,7970 | 2479 | 308 | ERS4338935 |
| 13CEB320STA | 2013 | Belgium | FPO | human | 5 | 2 | 2,7970 | 2478 | 346 | ERS4338936 |
| 13CEB322STA | 2013 | Belgium | FPO | human | 3 | 3 | 2,7712 | 2601 | 479 | ERS4338937 |
| 13CEB323STA | 2013 | Belgium | FPO | human | 72 | 2 | 2,7090 | 2671 | 359 | ERS4338938 |
| 13CEB324STA | 2013 | Belgium | FPO | human | 2383 | 5 | 2,7467 | 2533 | 357 | ERS4338939 |
| 13CEB326STA | 2013 | Belgium | FPO | human | 398 | 5 | 2,6868 | 2628 | 419 | ERS4338940 |
| 13CEB327STA | 2013 | Belgium | FPO | human | 34 | 10 | 2,8329 | 2549 | 279 | ERS4338941 |
| 13CEB328STA | 2013 | Belgium | FPO | human | 34 | 15 | 2,8082 | 2518 | 451 | ERS4338942 |
| 13CEB329STA | 2013 | Belgium | FPO | human | 5 | 7 | 2,7452 | 2495 | 412 | ERS4338943 |
| 13CEB332STA | 2013 | Belgium | FPO | dairy product | 133 | 13 | 2,7593 | 2318 | 292 | ERS4338944 |
| 13CEB412STA | 2013 | France | FPO | ready-to-eat food | 1 | 6 | 2,7502 | 2544 | 127 | ERS4338945 |
| 13CEB417STA | 2013 | France | FPO | meat | 1 | 3 | 2,7508 | 2539 | 370 | ERS4338946 |
| 13CEB422STA | 2013 | France | FPO | dairy product | 1 | 7 | 2,7520 | 2539 | 333 | ERS4338947 |
| 13CEB427STA | 2013 | France | FPO | meat | 1 | 6 | 2,7510 | 2546 | 371 | ERS4338948 |
| 13CEB52STA / NCTC8325 variant | 2013 | Italy | reference strain | NA | 8 | 1 | 2,7569 | 2751 | 308 | ERS4338949 |
| 14A / FRI361 | 1962 | United Kingdom | FPO | meat | 47 | 2 | 2,7233 | 2524 | 361 | ERS4338950 |
| 15SBCL1201STA | 2015 | Algeria | study | dairy product | 8 | 10 | 2,8145 | 2605 | 657 | ERS4338951 |
| 15SBCL1206STA | 2015 | Algeria | study | dairy product | 8 | 10 | 2,8122 | 2605 | 662 | ERS4338952 |
| 15SBCL1211STA | 2015 | Algeria | study | dairy product | 15 | 4 | 2,7007 | 2473 | 317 | ERS4338953 |
| 15SBCL1218STA | 2015 | Algeria | study | dairy product | 7 | 4 | 2,7308 | 2510 | 430 | ERS4338954 |
| 15SBCL1220STA | 2015 | Algeria | study | dairy product | 15 | 3 | 2,7020 | 2475 | 353 | ERS4338955 |
| 15SBCL1228STA | 2015 | Algeria | study | dairy product | 8 | 10 | 3,1103 | 2640 | 531 | ERS4338956 |
| 15SBCL1251STA | 2015 | Algeria | study | dairy product | 8 | 7 | 2,8150 | 2607 | 287 | ERS4338957 |
| 15SBCL1261STA | 2015 | Algeria | study | dairy product | 6 | 10 | 2,7080 | 2472 | 218 | ERS4338958 |
| 15SBCL1262STA | 2015 | Algeria | study | dairy product | 8 | 10 | 2,8162 | 2602 | 442 | ERS4338959 |
| 15SBCL1267STA | 2015 | Algeria | study | dairy product | 8 | 11 | 2,8136 | 2605 | 631 | ERS4338960 |
| 15SBCL1292STA | 2015 | France | FPO | dairy product | 389 | 8 | 2,7254 | 2505 | 446 | ERS4338961 |
| 15SBCL1299STA | 2015 | France | FPO | ready-to-eat food | 389 | 33 | 2,8199 | 2499 | 453 | ERS4338962 |
| 15SBCL1314STA | 2015 | Italy | FPO | dairy product | 30 | 5 | 2,7960 | 2605 | 190 | ERS4338963 |
| 15SBCL1391STA | 2015 | France | FPO | dairy product | 97 | 10 | 2,7163 | 2501 | 266 | ERS4338964 |
| 15SBCL1397STA | 2015 | France | FPO | dairy product | 389 | 14 | 2,7245 | 2496 | 502 | ERS4338965 |
| 15SBCL1404STA | 2015 | France | FPO | dairy product | 133 | 20 | 2,8421 | 2307 | 520 | ERS4338966 |
| 15SBCL1409STA | 2015 | France | FPO | dairy product | 389 | 12 | 2,7754 | 2556 | 467 | ERS4338967 |
| 15SBCL1428STA | 2015 | France | FPO | dairy product | 389 | 5 | 2,7261 | 2505 | 387 | ERS4338968 |
| 15SBCL1430STA | 2015 | France | FPO | dairy product | 389 | 6 | 2,7251 | 2500 | 379 | ERS4338969 |
| 15SBCL1434STA | 2015 | France | FPO | dairy product | 71 | 69 | 2,9395 | 2410 | 323 | ERS4338970 |
| 15SBCL1438STA | 2015 | France | FPO | dairy product | 504 | 2 | 2,7537 | 2600 | 389 | ERS4338971 |
| 15SBCL1506STA | 2015 | Ireland | FPO | ready-to-eat food | 8 | 4 | 2,8698 | 2704 | 210 | ERS4338972 |
| 15SBCL1507STA | 2015 | Ireland | FPO | meat | 5 | 1 | 2,7298 | 2514 | 263 | ERS4338973 |
| 15SBCL1509STA | 2015 | Ireland | FPO | meat | 7 | 3 | 2,7332 | 2575 | 255 | ERS4338974 |
| 15SBCL1517STA | 2015 | Ireland | FPO | meat | 22 | 1 | 2,7796 | 2585 | 292 | ERS4338975 |
| 15SBCL1520STA | 2015 | Ireland | FPO | ready-to-eat food | 1 | 2 | 2,7874 | 2583 | 346 | ERS4338976 |
| 15SBCL1527STA | 2015 | Ireland | FPO | ready-to-eat food | 22 | 4 | 2,7142 | 2491 | 353 | ERS4338977 |
| 15SBCL1540STA | 2015 | Ireland | FPO | ready-to-eat food | 1 | 4 | 2,7907 | 2580 | 275 | ERS4338978 |
| 15SBCL1548STA | 2015 | Ireland | FPO | ready-to-eat food | 1 | 4 | 2,7886 | 2579 | 262 | ERS4338979 |
| 15SBCL1550STA | 2015 | Ireland | FPO | ready-to-eat food | 5 | 1 | 2,7077 | 2475 | 335 | ERS4338980 |
| 15SBCL1560STA | 2015 | Cyprus | FPO | meat | 1 | 5 | 2,7221 | 2489 | 380 | ERS4338981 |
| 15SBCL1565STA | 2015 | Cyprus | FPO | meat | 1 | 4 | 2,7232 | 2490 | 363 | ERS4338982 |
| 15SBCL1570STA | 2015 | Cyprus | FPO | human | 1 | 5 | 2,7217 | 2491 | 382 | ERS4338983 |
| 15SBCL1571STA | 2015 | Cyprus | FPO | human | 1 | 1 | 2,7214 | 2534 | 301 | ERS4338984 |
| 15SBCL1576STA | 2015 | Cyprus | FPO | ready-to-eat food | 1 | 5 | 2,7280 | 2488 | 402 | ERS4338985 |
| 17SBCL03STA | 2017 | France | FPO | ready-to-eat food | 97 | 4 | 2,7865 | 2561 | 209 | ERS4338986 |
| 17SBCL07STA | 2017 | France | FPO | ready-to-eat food | CC97 | 3 | 2,7870 | 2560 | 174 | ERS4338987 |
| 17SBCL08STA | 2017 | France | FPO | meat | 5 | 7 | 2,8023 | 2454 | 246 | ERS4338988 |
| 17SBCL09STA | 2017 | France | FPO | meat | 5 | 3 | 2,7972 | 2456 | 179 | ERS4338989 |
| 17SBCL13STA | 2017 | France | FPO | meat | CC5 | 250 | 3,2513 | 2264 | 287 | ERS4338990 |
| 17SBCL18STA | 2017 | France | FPO | meat | 59 | 3 | 2,7064 | 2491 | 376 | ERS4338991 |
| 17SBCL202STA | 2017 | France | FPO | dairy product | 389 | 45 | 2,7308 | 2489 | 530 | ERS4338992 |
| 17SBCL208STA | 2017 | France | FPO | dairy product | 389 | 4 | 2,6536 | 2453 | 186 | ERS4338993 |
| 17SBCL214STA | 2017 | France | FPO | dairy product | 389 | 5 | 2,7359 | 2500 | 210 | ERS4338994 |
| 17SBCL220STA | 2017 | France | FPO | dairy product | 389 | 5 | 2,7359 | 2486 | 182 | ERS4338995 |
| 17SBCL223STA | 2017 | France | FPO | dairy product | 97 | 7 | 2,7178 | 2500 | 311 | ERS4338996 |
| 17SBCL225STA | 2017 | France | FPO | dairy product | 389 | 5 | 2,7373 | 2495 | 394 | ERS4338997 |
| 17SBCL532STA | 2017 | France | FPO | dairy product | 1 | 4 | 2,7907 | 2585 | 633 | ERS4338998 |
| 17SBCL533STA | 2017 | France | FPO | dairy product | 1 | 7 | 2,7936 | 2587 | 351 | ERS4338999 |
| 17SBCL580STA | 2017 | Bulgaria | FPO | ready-to-eat food | 5 | 3 | 2,8085 | 2601 | 155 | ERS4339000 |
| 17SBCL585STA | 2017 | Bulgaria | FPO | ready-to-eat food | 5 | 9 | 2,8106 | 2603 | 145 | ERS4339001 |
| 17SBCL586STA | 2017 | Bulgaria | FPO | ready-to-eat food | 5 | 3 | 2,8088 | 2605 | 273 | ERS4339002 |
| 17SBCL778STA | 2017 | France | self-testing | dairy product | 97 | 16 | 2,7458 | 2500 | 360 | ERS4339003 |
| 1A / NCTC8532 | 1935 | United Kingdom | reference strain | human | 5 | 1 | 2,6882 | 2518 | 308 | ERS4339004 |
| 294E / FRI196E | 2001 | United States of America | reference strain | meat | 8 | 3 | 2,7956 | 2698 | 442 | ERS4339005 |
| 333E / FRI184 | 2001 | NA | reference strain | environment | 97 | 1 | 2,7812 | 2491 | 263 | ERS4339006 |
| 367F / A900322 | 2001 | NA | reference strain | human | 5 | 7 | 2,7132 | 2489 | 395 | ERS4339007 |
| 36A / FRI569 | 2001 | NA | reference strain | human | 1 | 2 | 2,7375 | 2565 | 254 | ERS4339008 |
| 388F | 1987 | France | SFPO | ready-to-eat food | 8 | 1 | 2,7008 | 2648 | 238 | ERS4339009 |
| 42A / FRIS6 | 2001 | NA | reference strain | NA | CC8 | 3 | 2,7388 | 2528 | 345 | ERS4339010 |
| 43A / FRI137 | 2001 | NA | reference strain | human | 10 | 7 | 2,7899 | 2619 | 442 | ERS4339011 |
| 44A / FRI1230 | 2001 | NA | reference strain | dairy product | 12 | 4 | 2,7288 | 2562 | 302 | ERS4339012 |
| 45A / FRI1151M | 2001 | NA | reference strain | NA | 8 | 2 | 2,7159 | 2598 | 302 | ERS4339013 |
| 46A / FRI326 | 2001 | NA | reference strain | meat | 395 | 9 | 2,7078 | 2529 | 363 | ERS4339014 |
| 47A / FRI1169 | 2001 | NA | reference strain | human | 8 | 4 | 2,7681 | 2619 | 239 | ERS4339015 |

Table SI : Uniprot accession number of SE protein sequences used as reference in genomic analyses

| Locus | Accession number of protein sequences |
| --- | --- |
| *sea* | P0A0L2 * |
| *seb* | P01552 * |
| *sec* | P01553 * |
| *sed* | P20723 * |
| *see* | P12993 * |
| *seg* | P0A0L8* |
| *seh* | P0A0L9 * |
| *sei* | [Q9KJX8](https://www.uniprot.org/uniprot/Q9KJX8) |
| *selj* | Q76LS7 |
| *sek* | A0A0H2XJE8 |
| *sel* | Q9F0L7 |
| *sem* | A0A0U1MQQ4 |
| *sen* | A5JJ08 |
| *seo* | A0A162H0T9 |
| *sep* | A0A075M4C7 |
| *seq* | Q93CC6 |
| *ser* | Q76LS8 |
| *ses* | B0I1V7 |
| *set* | B0I1V6 |
| *selu* | A0A5F0HMJ4 |
| *selv* | A0FKY6 |
| *selx* | G0Z026 |
| *sely* | A0A0K2S2V0 |
| *selz* | A0A5P6A8A0 |
| *sel26* | A0A2R4NHR4 |
| *sel27* | A0A2R4NHP6 |
| *TSST1* | P06886 * |
|  |  |
| * reviewed sequences in the UniProt database | |


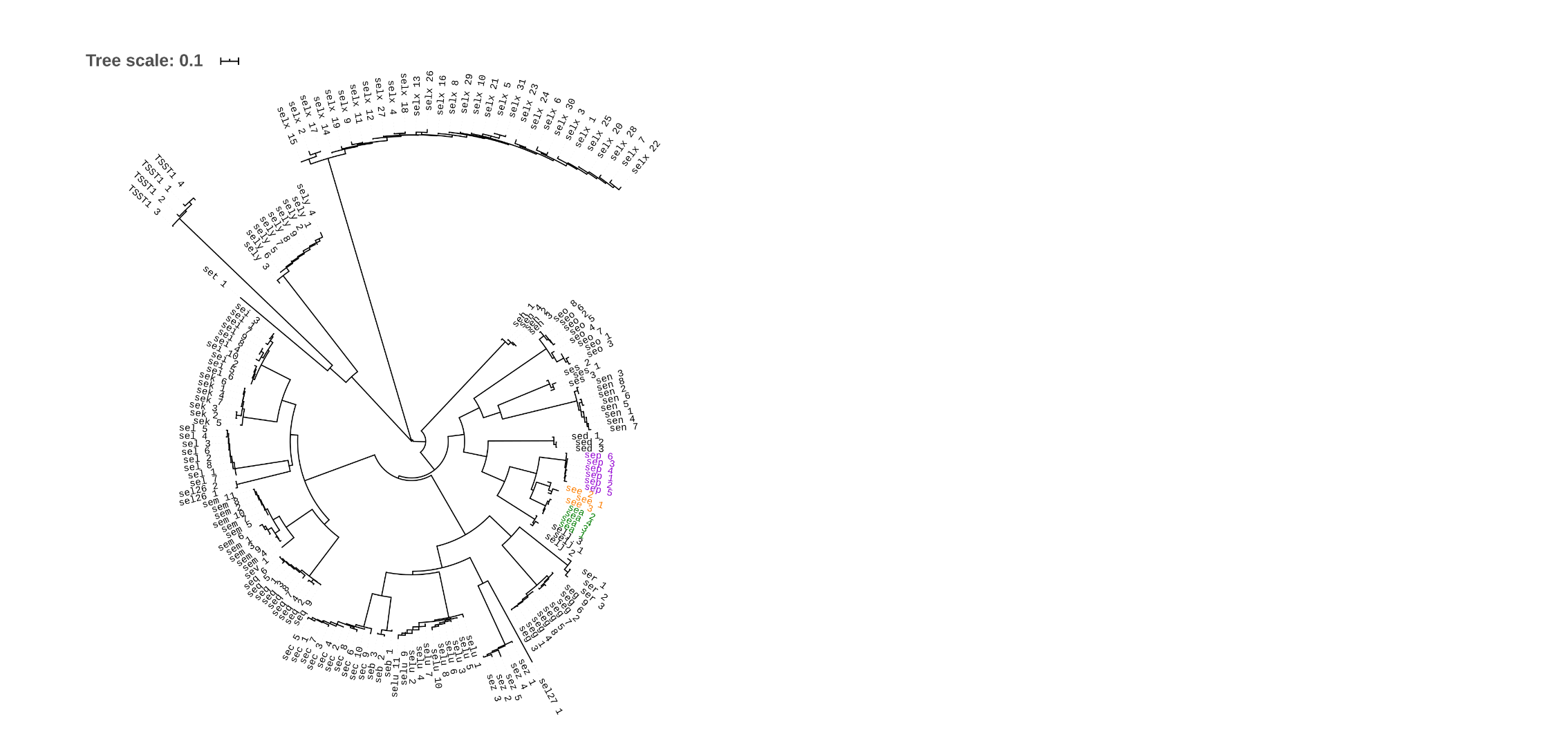

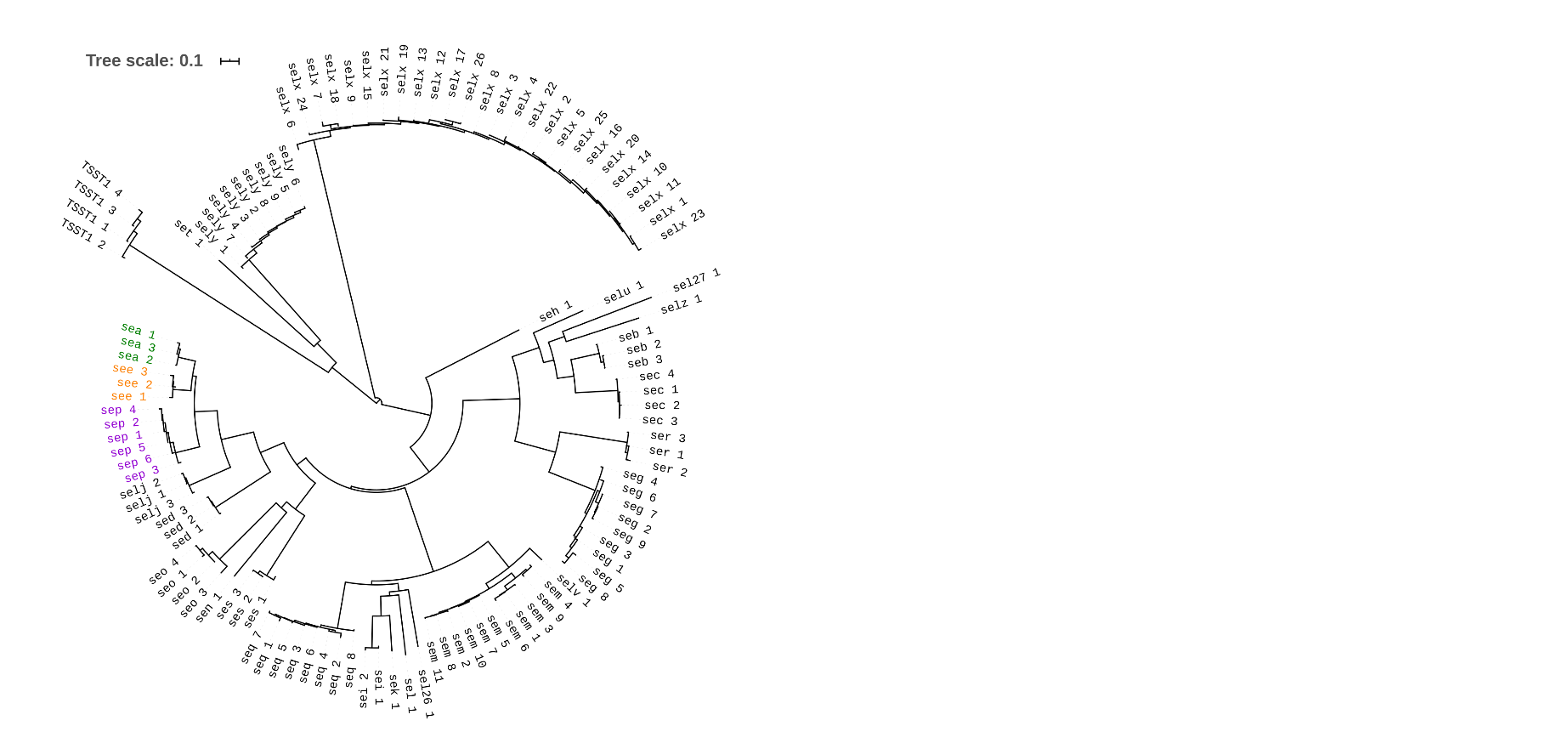

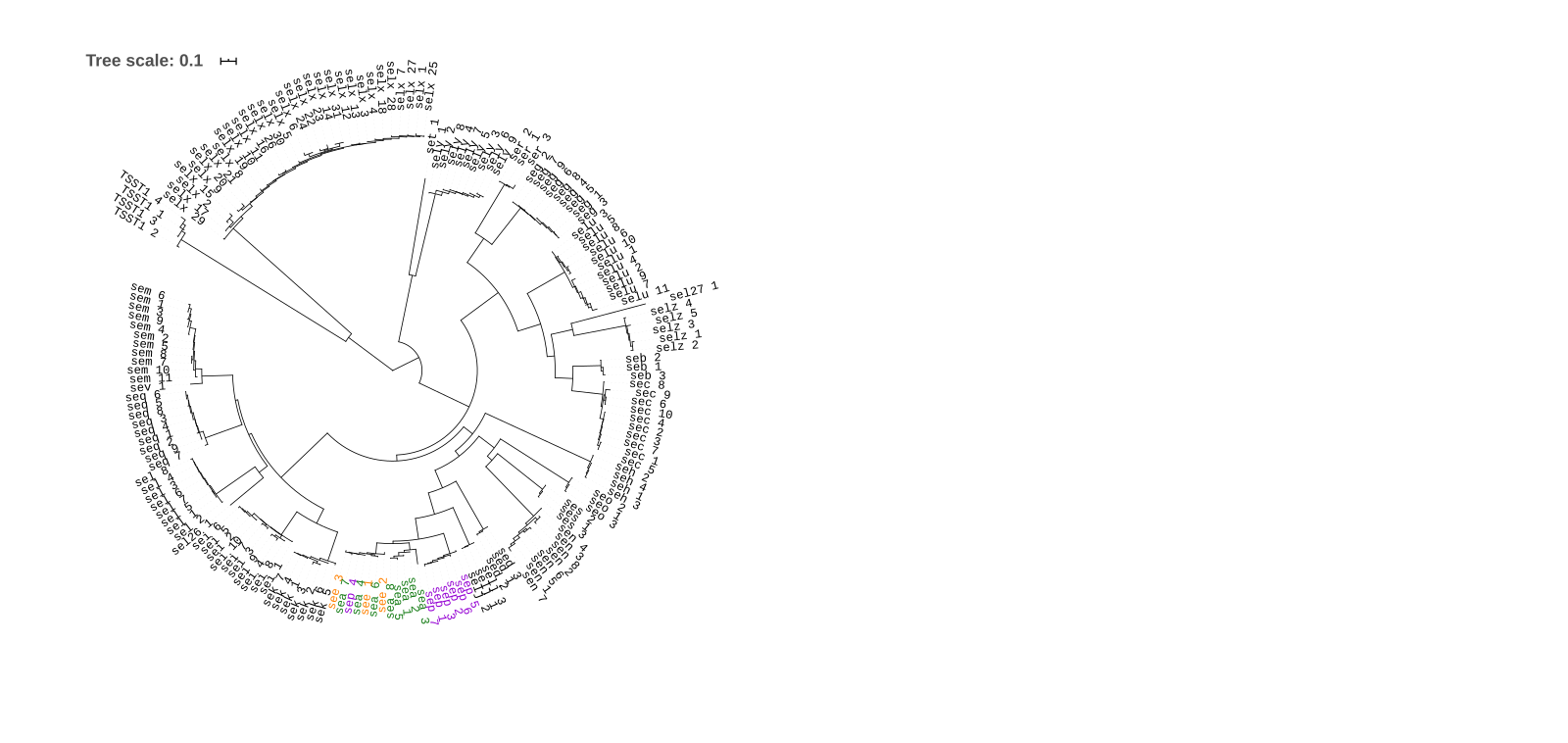


B.

C.

A.

Figure S1: Phylogenetic trees of protein variants obtained by NAuRA analyses. A. Phylogeny in neighbour joining obtained from 176 variants with released parameters (inferior to 84% of identity) for the three genes, *sea* (in green), *see* (in orange) and *sep* (in purple). B. Phylogeny in neighbour joining obtained from 114 variants with stringent parameters (superior to 84% of identity). C. Phylogeny in neighbour joining obtained from 177 variants with selected parameters for SE detection.

Table SIII: Matrix of presence or absence of SE by analyzed genomic sequences. 0: absence of SE genes. Before the dot, the protein variant number is indicated and after the dot, the nucleic variant number is indicated

|  | *sea* | *seb* | *sec* | *sed* | *see* | *seg* | *seh* | *sei* | *selj* | *sek* | *sel* | *sem* | *sen* | *seo* | *sep* | *seq* | *ser* | *ses* | *set* | *selu* | *selv* | *selx* | *sely* | *selz* | *sel26* | *sel27* | *TSST1* |
| --- | --- | --- | --- | --- | --- | --- | --- | --- | --- | --- | --- | --- | --- | --- | --- | --- | --- | --- | --- | --- | --- | --- | --- | --- | --- | --- | --- |
| 05CEB01STA | 3.1 | 0 | 2.3 | 0 | 0 | 0 | 0 | 0 | 0 | 0 | 2.3 | 0 | 0 | 0 | 0 | 0 | 0 | 0 | 0 | 0 | 0 | 29.2 | 0 | 0 | 0 | 0 | 2.3 |
| 05CEB18STA | 2.7 | 0 | 0 | 0 | 0 | 0 | 2.3 | 0 | 0 | 5.5 | 0 | 0 | 0 | 0 | 0 | 4.1 | 0 | 0 | 0 | 0 | 0 | 6.2 | 0 | 0 | 0 | 0 | 1.4 |
| 05CEB52STA | 0 | 3.1 | 0 | 0 | 0 | 4.1 | 0 | 4.1 | 0 | 0 | 0 | 4.1 | 4.1 | 4.1 | 0 | 0 | 0 | 0 | 0 | 3.1 | 0 | 5.3 | 8.2 | 2.1 | 0 | 0 | 0 |
| 07CEB132STA | 0 | 0 | 0 | 1.3 | 0 | 0 | 0 | 0 | 1.4 | 0 | 0 | 0 | 0 | 0 | 0 | 0 | 1.8 | 0 | 0 | 0 | 0 | 7.1 | 0 | 0 | 0 | 0 | 0 |
| 07CEB151STA | 0 | 0 | 0 | 0 | 0 | 0 | 0 | 0 | 0 | 0 | 0 | 0 | 0 | 0 | 0 | 0 | 0 | 0 | 0 | 0 | 0 | 12.4 | 0 | 1.4 | 0 | 0 | 0 |
| 07CEB153STA | 0 | 0 | 2.1 | 0 | 0 | 0 | 0 | 0 | 0 | 0 | 2.1 | 0 | 0 | 0 | 0 | 0 | 0 | 0 | 0 | 0 | 0 | 8.3 | 0 | 0 | 0 | 0 | 2.2 |
| 07CEB234STA | 0 | 0 | 0 | 0 | 0 | 0 | 0 | 0 | 0 | 0 | 0 | 0 | 0 | 0 | 0 | 0 | 0 | 0 | 0 | 0 | 0 | 5.2 | 0 | 0 | 0 | 0 | 0 |
| 07CEB89STA | 2.7 | 0 | 0 | 0 | 0 | 0 | 0 | 0 | 0 | 0 | 0 | 0 | 0 | 0 | 0 | 0 | 0 | 0 | 0 | 0 | 0 | 3.7 | 0 | 0 | 0 | 0 | 0 |
| 07CEB90STA | 0 | 0 | 6.3 | 0 | 0 | 2.24 | 0 | 6.1 | 0 | 0 | 4.1 | 7.1 | 2.28 | 2.13 | 0 | 0 | 0 | 0 | 0 | 7.6 | 0 | 0 | 0 | 0 | 0 | 0 | 0 |
| 07CEB91STA | 0 | 0 | 0 | 0 | 0 | 0 | 0 | 0 | 0 | 0 | 0 | 0 | 0 | 0 | 3.14 | 0 | 0 | 0 | 0 | 0 | 0 | 7.16 | 0 | 0 | 0 | 0 | 0 |
| 07CEB93STA | 0 | 0 | 0 | 0 | 0 | 0 | 0 | 0 | 0 | 0 | 0 | 0 | 0 | 0 | 0 | 0 | 0 | 0 | 0 | 0 | 0 | 1.2 | 0 | 0 | 0 | 0 | 0 |
| 07CEB94STA | 1.2 | 0 | 0 | 0 | 0 | 2.13 | 0 | 2.14 | 0 | 0 | 0 | 2.13 | 2.19 | 0 | 0 | 0 | 0 | 0 | 0 | 0 | 0 | 4.2 | 0 | 0 | 0 | 0 | 0 |
| 09CEB04STA | 0 | 0 | 0 | 0 | 0 | 0 | 0 | 0 | 0 | 0 | 0 | 0 | 0 | 0 | 0 | 0 | 0 | 0 | 0 | 0 | 0 | 7.5 | 0 | 0 | 0 | 0 | 0 |
| 09CEB204STA | 3.1 | 0 | 0 | 0 | 0 | 0 | 0 | 0 | 0 | 0 | 0 | 0 | 0 | 0 | 0 | 0 | 0 | 0 | 0 | 0 | 0 | 21.1 | 0 | 0 | 0 | 0 | 0 |
| 09CEB303STA | 0 | 0 | 0 | 0 | 0 | 0 | 0 | 0 | 2.1 | 0 | 0 | 0 | 0 | 0 | 0 | 0 | 4.2 | 1.1 | 1.1 | 0 | 0 | 7.7 | 0 | 0 | 0 | 0 | 0 |
| 09CEB314STA | 0 | 0 | 0 | 0 | 2.3 | 0 | 0 | 0 | 0 | 0 | 0 | 0 | 0 | 0 | 0 | 0 | 0 | 0 | 0 | 0 | 0 | 14.3 | 0 | 0 | 0 | 0 | 0 |
| 09CEB319STA | 0 | 0 | 0 | 0 | 2.2 | 0 | 0 | 0 | 0 | 0 | 0 | 0 | 0 | 0 | 0 | 0 | 0 | 0 | 0 | 0 | 0 | 14.1 | 0 | 0 | 0 | 0 | 0 |
| 09CEB329STA | 0 | 0 | 0 | 0 | 2.1 | 0 | 0 | 0 | 0 | 0 | 0 | 0 | 0 | 0 | 0 | 0 | 0 | 0 | 0 | 0 | 0 | 14.4 | 0 | 0 | 0 | 0 | 0 |
| 11CEB145STA | 2.1 | 0 | 9.1 | 0 | 0 | 2.28 | 0 | 2.22 | 0 | 0 | 1.6 | 2.25 | 2.27 | 2.16 | 0 | 0 | 0 | 0 | 0 | 0 | 0 | 4.11 | 0 | 0 | 0 | 0 | 1.7 |
| 11CEB272STA | 2.17 | 0 | 0 | 1.4 | 0 | 0 | 0 | 0 | 1.2 | 0 | 0 | 0 | 0 | 0 | 0 | 0 | 3.2 | 0 | 0 | 0 | 0 | 7.2 | 0 | 0 | 0 | 0 | 0 |
| 11CEB273STA | 0 | 0 | 0 | 1.1 | 0 | 0 | 0 | 0 | 1.1 | 0 | 0 | 0 | 0 | 0 | 0 | 0 | 1.1 | 0 | 0 | 0 | 0 | 7.6 | 0 | 0 | 0 | 0 | 0 |
| 11CEB274STA | 0 | 0 | 0 | 0 | 0 | 0 | 0 | 0 | 0 | 0 | 0 | 0 | 0 | 0 | 0 | 0 | 0 | 0 | 0 | 0 | 0 | 12.6 | 0 | 1.4 | 0 | 0 | 0 |
| 11CEB275STA | 0 | 0 | 0 | 0 | 0 | 0 | 0 | 0 | 0 | 0 | 0 | 0 | 0 | 0 | 0 | 0 | 0 | 0 | 0 | 0 | 0 | 12.3 | 0 | 1.2 | 0 | 0 | 0 |
| 11CEB276STA | 0 | 0 | 0 | 1.7 | 0 | 0 | 0 | 0 | 1.6 | 0 | 0 | 0 | 0 | 0 | 0 | 0 | 1.7 | 0 | 0 | 0 | 0 | 7.1 | 0 | 0 | 0 | 0 | 0 |
| 11CEB277STA | 0 | 0 | 0 | 0 | 0 | 2.27 | 0 | 2.24 | 0 | 0 | 0 | 2.17 | 2.24 | 2.24 | 0 | 0 | 0 | 0 | 0 | 2.9 | 0 | 2.5 | 1.5 | 0 | 0 | 0 | 0 |
| 11CEB278STA | 0 | 0 | 0 | 1.8 | 0 | 0 | 0 | 0 | 1.7 | 0 | 0 | 0 | 0 | 0 | 0 | 0 | 1.6 | 0 | 0 | 0 | 0 | 7.9 | 0 | 0 | 0 | 0 | 0 |
| 11CEB279STA | 0 | 0 | 0 | 0 | 0 | 2.12 | 0 | 2.9 | 0 | 0 | 0 | 2.12 | 2.11 | 2.12 | 0 | 0 | 0 | 0 | 0 | 2.5 | 0 | 2.6 | 1.5 | 0 | 0 | 0 | 0 |
| 11CEB280STA | 0 | 0 | 0 | 0 | 0 | 0 | 0 | 0 | 0 | 0 | 0 | 0 | 0 | 0 | 0 | 0 | 0 | 0 | 0 | 0 | 0 | 12.9 | 0 | 1.4 | 0 | 0 | 0 |
| 11CEB281STA | 0 | 0 | 0 | 1.1 | 0 | 0 | 0 | 0 | 1.1 | 0 | 0 | 0 | 0 | 0 | 0 | 0 | 1.1 | 0 | 0 | 0 | 0 | 7.13 | 0 | 0 | 0 | 0 | 0 |
| 11CEB282STA | 2.2 | 0 | 0 | 1.5 | 0 | 0 | 0 | 0 | 1.3 | 0 | 0 | 0 | 0 | 0 | 0 | 0 | 3.1 | 0 | 0 | 0 | 0 | 7.20 | 0 | 0 | 0 | 0 | 0 |
| 11CEB283STA | 0 | 0 | 0 | 0 | 0 | 0 | 0 | 0 | 0 | 0 | 0 | 0 | 0 | 0 | 0 | 0 | 0 | 0 | 0 | 0 | 0 | 12.1 | 0 | 1.4 | 0 | 0 | 0 |
| 11CEB284STA | 0 | 0 | 0 | 1.2 | 0 | 0 | 0 | 0 | 1.10 | 0 | 0 | 0 | 0 | 0 | 0 | 0 | 1.3 | 0 | 0 | 0 | 0 | 7.18 | 0 | 0 | 0 | 0 | 0 |
| 11CEB285STA | 0 | 0 | 0 | 0 | 0 | 0 | 0 | 0 | 0 | 0 | 0 | 0 | 0 | 0 | 0 | 0 | 0 | 0 | 0 | 0 | 0 | 12.7 | 0 | 1.5 | 0 | 0 | 0 |
| 13CEB175STA | 2.15 | 0 | 0 | 1.6 | 0 | 0 | 0 | 0 | 1.5 | 0 | 0 | 0 | 0 | 0 | 0 | 0 | 1.2 | 0 | 0 | 0 | 0 | 7.11 | 0 | 0 | 0 | 0 | 0 |
| 13CEB176STA | 0 | 1.5 | 0 | 0 | 0 | 0 | 0 | 0 | 0 | 1.4 | 0 | 0 | 0 | 0 | 0 | 1.3 | 0 | 0 | 0 | 0 | 0 | 7.3 | 0 | 0 | 0 | 0 | 0 |
| 13CEB177STA | 0 | 0 | 1.2 | 0 | 0 | 1.1 | 1.3 | 1.1 | 0 | 0 | 1.7 | 1.1 | 1.1 | 1.1 | 0 | 0 | 0 | 0 | 0 | 5.1 | 0 | 10.1 | 0 | 0 | 0 | 0 | 0 |
| 13CEB178STA | 0 | 0 | 0 | 2.1 | 0 | 2.21 | 0 | 2.20 | 1.8 | 0 | 0 | 2.4 | 2.22 | 2.4 | 0 | 0 | 1.8 | 0 | 0 | 0 | 0 | 4.7 | 0 | 0 | 0 | 0 | 0 |
| 13CEB179STA | 0 | 0 | 5.2 | 0 | 0 | 2.19 | 0 | 10.4 | 0 | 0 | 1.7 | 8.4 | 2.18 | 2.18 | 0 | 0 | 0 | 0 | 0 | 9.2 | 0 | 17.3 | 0 | 0 | 0 | 0 | 0 |
| 13CEB181STA | 1.5 | 0 | 0 | 0 | 0 | 3.1 | 1.1 | 3.2 | 0 | 0 | 0 | 3.2 | 3.1 | 3.2 | 0 | 0 | 0 | 0 | 0 | 1.1 | 0 | 0 | 0 | 0 | 0 | 0 | 4.4 |
| 13CEB182STA | 0 | 0 | 4.3 | 0 | 0 | 0 | 0 | 8.3 | 0 | 0 | 1.8 | 0 | 7.4 | 8.4 | 0 | 0 | 0 | 0 | 0 | 8.3 | 0 | 14.5 | 7.1 | 1.3 | 0 | 0 | 3.2 |
| 13CEB184STA | 0 | 0 | 0 | 0 | 0 | 0 | 2.7 | 0 | 0 | 0 | 0 | 0 | 0 | 0 | 0 | 0 | 0 | 0 | 0 | 0 | 0 | 6.4 | 0 | 0 | 0 | 0 | 0 |
| 13CEB188STA | 0 | 0 | 0 | 0 | 0 | 2.18 | 0 | 5.2 | 0 | 0 | 0 | 2.15 | 2.15 | 2.15 | 3.6 | 0 | 0 | 0 | 0 | 0 | 0 | 4.14 | 0 | 0 | 0 | 0 | 0 |
| 13CEB190STA | 0 | 0 | 4.1 | 0 | 0 | 0 | 0 | 8.1 | 0 | 0 | 1.3 | 0 | 7.1 | 8.2 | 0 | 0 | 0 | 0 | 0 | 8.2 | 0 | 14.6 | 7.3 | 1.1 | 0 | 0 | 3.1 |
| 13CEB191STA | 0 | 0 | 0 | 2.1 | 0 | 2.17 | 0 | 2.1 | 1.8 | 0 | 0 | 2.3 | 2.14 | 2.3 | 2.4 | 0 | 1.8 | 0 | 0 | 0 | 0 | 4.10 | 0 | 0 | 0 | 0 | 0 |
| 13CEB193STA | 3.2 | 0 | 2.1 | 0 | 0 | 0 | 0 | 0 | 0 | 0 | 2.1 | 0 | 0 | 0 | 0 | 0 | 0 | 0 | 0 | 0 | 0 | 8.3 | 0 | 0 | 0 | 0 | 2.2 |
| 13CEB235STA | 2.22 | 0 | 0 | 0 | 0 | 0 | 0 | 0 | 0 | 0 | 0 | 0 | 0 | 0 | 0 | 0 | 0 | 0 | 0 | 0 | 0 | 3.2 | 0 | 0 | 0 | 0 | 0 |
| 13CEB239STA | 2.2 | 0 | 0 | 0 | 0 | 0 | 0 | 0 | 0 | 0 | 0 | 0 | 0 | 0 | 0 | 0 | 0 | 0 | 0 | 0 | 0 | 3.3 | 0 | 0 | 0 | 0 | 0 |
| 13CEB243STA | 2.11 | 0 | 0 | 0 | 0 | 0 | 0 | 0 | 0 | 0 | 0 | 0 | 0 | 0 | 0 | 0 | 0 | 0 | 0 | 0 | 0 | 3.5 | 0 | 0 | 0 | 0 | 0 |
| 13CEB251STA | 0 | 0 | 3.2 | 0 | 0 | 0 | 0 | 0 | 0 | 0 | 3.2 | 0 | 0 | 0 | 0 | 0 | 0 | 0 | 0 | 0 | 0 | 5.1 | 0 | 0 | 0 | 0 | 2.6 |
| 13CEB257STA | 0 | 0 | 3.1 | 0 | 0 | 0 | 0 | 0 | 0 | 0 | 3.1 | 0 | 0 | 0 | 0 | 0 | 0 | 0 | 0 | 0 | 0 | 5.4 | 0 | 0 | 0 | 0 | 2.1 |
| 13CEB307STA | 2.5 | 0 | 8.2 | 0 | 0 | 0 | 0 | 0 | 0 | 5.3 | 6.2 | 0 | 0 | 0 | 0 | 4.2 | 0 | 0 | 0 | 0 | 0 | 7.7 | 0 | 0 | 0 | 0 | 0 |
| 13CEB308STA | 2.7 | 0 | 0 | 0 | 0 | 0 | 0 | 0 | 0 | 0 | 0 | 0 | 0 | 0 | 0 | 0 | 0 | 0 | 0 | 0 | 0 | 3.6 | 0 | 0 | 0 | 0 | 0 |
| 13CEB309STA | 2.20 | 0 | 0 | 0 | 0 | 0 | 0 | 0 | 0 | 0 | 0 | 0 | 0 | 0 | 0 | 0 | 0 | 0 | 0 | 0 | 0 | 3.4 | 0 | 0 | 0 | 0 | 0 |
| 13CEB310STA | 2.1 | 0 | 8.1 | 0 | 0 | 0 | 0 | 0 | 0 | 5.1 | 6.1 | 0 | 0 | 0 | 0 | 4.6 | 0 | 0 | 0 | 0 | 0 | 7.1 | 0 | 0 | 0 | 0 | 0 |
| 13CEB311STA | 0 | 0 | 0 | 0 | 0 | 0 | 0 | 0 | 0 | 0 | 0 | 0 | 0 | 0 | 2.2 | 0 | 0 | 0 | 0 | 0 | 0 | 1.1 | 0 | 0 | 0 | 0 | 0 |
| 13CEB312STA | 1.3 | 0 | 0 | 0 | 0 | 3.6 | 0 | 3.10 | 0 | 0 | 0 | 3.8 | 3.8 | 3.9 | 0 | 0 | 0 | 0 | 0 | 1.10 | 0 | 0 | 0 | 0 | 0 | 0 | 0 |
| 13CEB313STA | 1.3 | 0 | 0 | 0 | 0 | 3.5 | 0 | 3.6 | 0 | 0 | 0 | 3.7 | 3.6 | 3.7 | 0 | 0 | 0 | 0 | 0 | 1.5 | 0 | 0 | 0 | 0 | 0 | 0 | 0 |
| 13CEB314STA | 1.4 | 0 | 0 | 0 | 0 | 3.3 | 0 | 3.1 | 0 | 0 | 0 | 3.3 | 3.3 | 3.4 | 0 | 0 | 0 | 0 | 0 | 1.3 | 0 | 0 | 0 | 0 | 0 | 0 | 0 |
| 13CEB315STA | 1.7 | 0 | 0 | 0 | 0 | 3.2 | 0 | 3.5 | 0 | 0 | 0 | 3.4 | 3.4 | 3.5 | 0 | 0 | 0 | 0 | 0 | 1.4 | 0 | 0 | 0 | 0 | 0 | 0 | 0 |
| 13CEB316STA | 1.6 | 0 | 0 | 0 | 0 | 3.10 | 0 | 3.8 | 0 | 0 | 0 | 3.12 | 3.11 | 3.12 | 0 | 0 | 0 | 0 | 0 | 1.11 | 0 | 0 | 0 | 0 | 0 | 0 | 0 |
| 13CEB317STA | 1.3 | 0 | 0 | 0 | 0 | 3.4 | 0 | 3.11 | 0 | 0 | 0 | 3.5 | 3.5 | 3.6 | 0 | 0 | 0 | 0 | 0 | 1.6 | 0 | 0 | 0 | 0 | 0 | 0 | 0 |
| 13CEB318STA | 1.3 | 0 | 0 | 0 | 0 | 3.9 | 0 | 3.7 | 0 | 0 | 0 | 3.6 | 3.7 | 3.8 | 0 | 0 | 0 | 0 | 0 | 1.7 | 0 | 0 | 0 | 0 | 0 | 0 | 0 |
| 13CEB319STA | 2.7 | 0 | 0 | 0 | 0 | 6.2 | 0 | 2.9 | 1.8 | 0 | 0 | 2.12 | 2.11 | 2.12 | 0 | 0 | 1.8 | 0 | 0 | 0 | 0 | 4.15 | 0 | 0 | 0 | 0 | 0 |
| 13CEB320STA | 2.20 | 0 | 0 | 0 | 0 | 6.1 | 0 | 2.23 | 1.8 | 0 | 0 | 2.23 | 2.3 | 2.23 | 0 | 0 | 1.8 | 0 | 0 | 0 | 0 | 4.4 | 0 | 0 | 0 | 0 | 0 |
| 13CEB322STA | 2.7 | 0 | 0 | 0 | 0 | 0 | 2.1 | 0 | 0 | 5.5 | 0 | 0 | 0 | 0 | 0 | 4.1 | 0 | 0 | 0 | 0 | 0 | 6.7 | 0 | 0 | 0 | 0 | 0 |
| 13CEB323STA | 0 | 0 | 7.1 | 0 | 0 | 7.2 | 0 | 2.11 | 0 | 0 | 5.1 | 2.14 | 6.1 | 7.3 | 0 | 0 | 0 | 0 | 0 | 7.2 | 0 | 6.8 | 0 | 0 | 0 | 0 | 0 |
| 13CEB324STA | 2.8 | 0 | 0 | 0 | 0 | 0 | 2.3 | 0 | 0 | 5.10 | 0 | 0 | 0 | 0 | 0 | 4.10 | 0 | 0 | 0 | 0 | 0 | 6.6 | 0 | 0 | 0 | 0 | 0 |
| 13CEB326STA | 0 | 0 | 0 | 0 | 0 | 0 | 0 | 0 | 0 | 0 | 0 | 0 | 0 | 0 | 0 | 0 | 0 | 0 | 0 | 0 | 0 | 0 | 0 | 0 | 0 | 0 | 0 |
| 13CEB327STA | 0 | 0 | 0 | 0 | 0 | 3.7 | 1.2 | 3.4 | 0 | 0 | 0 | 3.1 | 3.10 | 3.3 | 0 | 0 | 0 | 0 | 0 | 1.8 | 0 | 0 | 0 | 0 | 0 | 0 | 4.4 |
| 13CEB328STA | 0 | 0 | 0 | 0 | 0 | 3.8 | 1.5 | 3.9 | 0 | 0 | 0 | 3.9 | 3.9 | 3.10 | 0 | 0 | 0 | 0 | 0 | 1.9 | 0 | 0 | 0 | 0 | 0 | 0 | 4.2 |
| 13CEB329STA | 0 | 0 | 0 | 0 | 0 | 2.15 | 0 | 2.11 | 0 | 0 | 0 | 2.14 | 2.16 | 2.17 | 0 | 0 | 0 | 0 | 0 | 0 | 0 | 4.3 | 0 | 0 | 0 | 0 | 0 |
| 13CEB332STA | 0 | 0 | 2.5 | 0 | 0 | 0 | 0 | 0 | 0 | 0 | 2.5 | 0 | 0 | 0 | 0 | 0 | 0 | 0 | 0 | 0 | 0 | 8.1 | 0 | 0 | 0 | 0 | 2.4 |
| 13CEB412STA | 2.22 | 0 | 0 | 0 | 0 | 0 | 2.2 | 0 | 0 | 5.4 | 0 | 0 | 0 | 0 | 0 | 4.5 | 0 | 0 | 0 | 0 | 0 | 6.2 | 0 | 0 | 0 | 0 | 1.4 |
| 13CEB417STA | 2.19 | 0 | 0 | 0 | 0 | 0 | 2.3 | 0 | 0 | 5.6 | 0 | 0 | 0 | 0 | 0 | 4.7 | 0 | 0 | 0 | 0 | 0 | 6.7 | 0 | 0 | 0 | 0 | 1.2 |
| 13CEB422STA | 2.12 | 0 | 0 | 0 | 0 | 0 | 2.3 | 0 | 0 | 5.8 | 0 | 0 | 0 | 0 | 0 | 4.9 | 0 | 0 | 0 | 0 | 0 | 6.10 | 0 | 0 | 0 | 0 | 1.1 |
| 13CEB427STA | 2.7 | 0 | 0 | 0 | 0 | 0 | 2.3 | 0 | 0 | 5.5 | 0 | 0 | 0 | 0 | 0 | 4.1 | 0 | 0 | 0 | 0 | 0 | 6.11 | 0 | 0 | 0 | 0 | 1.5 |
| 13CEB52STA | 0 | 0 | 0 | 0 | 0 | 0 | 0 | 0 | 0 | 0 | 0 | 0 | 0 | 0 | 0 | 0 | 0 | 0 | 0 | 0 | 0 | 7.4 | 0 | 0 | 0 | 0 | 0 |
| 14A | 0 | 0 | 6.1 | 1.9 | 0 | 2.15 | 0 | 6.2 | 1.9 | 0 | 7.1 | 5.2 | 2.16 | 2.17 | 0 | 0 | 1.4 | 0 | 0 | 7.4 | 0 | 0 | 0 | 0 | 0 | 0 | 0 |
| 15SBCL1201STA | 0 | 1.6 | 0 | 0 | 0 | 0 | 0 | 0 | 0 | 0 | 0 | 0 | 0 | 0 | 3.13 | 0 | 0 | 0 | 0 | 0 | 0 | 7.20 | 0 | 0 | 0 | 0 | 0 |
| 15SBCL1206STA | 0 | 1.9 | 0 | 0 | 0 | 0 | 0 | 0 | 0 | 0 | 0 | 0 | 0 | 0 | 3.5 | 0 | 0 | 0 | 0 | 0 | 0 | 7.15 | 0 | 0 | 0 | 0 | 0 |
| 15SBCL1211STA | 0 | 0 | 0 | 0 | 0 | 0 | 0 | 0 | 0 | 0 | 0 | 0 | 0 | 0 | 0 | 0 | 0 | 0 | 0 | 0 | 0 | 24.2 | 0 | 0 | 0 | 0 | 0 |
| 15SBCL1218STA | 0 | 0 | 0 | 0 | 0 | 0 | 0 | 0 | 0 | 0 | 0 | 0 | 0 | 0 | 2.3 | 0 | 0 | 0 | 0 | 0 | 0 | 1.2 | 0 | 0 | 0 | 0 | 0 |
| 15SBCL1220STA | 0 | 0 | 0 | 0 | 0 | 0 | 0 | 0 | 0 | 0 | 0 | 0 | 0 | 0 | 0 | 0 | 0 | 0 | 0 | 0 | 0 | 24.2 | 0 | 0 | 0 | 0 | 0 |
| 15SBCL1228STA | 0 | 1.1 | 0 | 0 | 0 | 0 | 0 | 0 | 0 | 0 | 0 | 0 | 0 | 0 | 3.8 | 0 | 0 | 0 | 0 | 0 | 0 | 7.19 | 0 | 0 | 0 | 0 | 0 |
| 15SBCL1251STA | 0 | 1.8 | 0 | 0 | 0 | 0 | 0 | 0 | 0 | 0 | 0 | 0 | 0 | 0 | 3.9 | 0 | 0 | 0 | 0 | 0 | 0 | 7.7 | 0 | 0 | 0 | 0 | 0 |
| 15SBCL1261STA | 0 | 0 | 0 | 0 | 0 | 0 | 0 | 0 | 0 | 0 | 0 | 0 | 0 | 0 | 0 | 0 | 0 | 0 | 0 | 0 | 0 | 3.1 | 0 | 0 | 0 | 0 | 0 |
| 15SBCL1262STA | 0 | 1.4 | 0 | 0 | 0 | 0 | 0 | 0 | 0 | 0 | 0 | 0 | 0 | 0 | 3.1 | 0 | 0 | 0 | 0 | 0 | 0 | 7.17 | 0 | 0 | 0 | 0 | 0 |
| 15SBCL1267STA | 0 | 1.3 | 0 | 0 | 0 | 0 | 0 | 0 | 0 | 0 | 0 | 0 | 0 | 0 | 3.11 | 0 | 0 | 0 | 0 | 0 | 0 | 7.23 | 0 | 0 | 0 | 0 | 0 |
| 15SBCL1292STA | 0 | 0 | 0 | 0 | 0 | 2.5 | 0 | 2.5 | 0 | 0 | 0 | 2.11 | 2.5 | 2.11 | 0 | 0 | 0 | 0 | 0 | 2.3 | 0 | 2.10 | 1.4 | 0 | 0 | 0 | 0 |
| 15SBCL1299STA | 0 | 0 | 0 | 0 | 0 | 2.20 | 0 | 2.18 | 0 | 0 | 0 | 2.22 | 2.21 | 2.22 | 0 | 0 | 0 | 0 | 0 | 2.8 | 0 | 2.9 | 1.3 | 0 | 0 | 0 | 0 |
| 15SBCL1314STA | 1.8 | 0 | 0 | 0 | 0 | 0 | 0 | 3.3 | 0 | 0 | 0 | 3.10 | 3.2 | 3.1 | 0 | 0 | 0 | 0 | 0 | 1.2 | 0 | 0 | 0 | 0 | 0 | 0 | 4.5 |
| 15SBCL1391STA | 0 | 0 | 0 | 0 | 0 | 0 | 0 | 0 | 0 | 0 | 0 | 0 | 0 | 0 | 0 | 0 | 0 | 0 | 0 | 0 | 0 | 12.8 | 0 | 0 | 0 | 0 | 0 |
| 15SBCL1397STA | 0 | 0 | 0 | 0 | 0 | 2.10 | 0 | 2.7 | 0 | 0 | 0 | 2.10 | 2.10 | 2.9 | 0 | 0 | 0 | 0 | 0 | 2.4 | 0 | 2.8 | 1.7 | 0 | 0 | 0 | 0 |
| 15SBCL1404STA | 0 | 0 | 2.4 | 0 | 0 | 0 | 0 | 0 | 0 | 0 | 2.4 | 0 | 0 | 0 | 0 | 0 | 0 | 0 | 0 | 0 | 0 | 8.2 | 0 | 0 | 0 | 0 | 2.5 |
| 15SBCL1409STA | 0 | 0 | 0 | 0 | 0 | 2.28 | 0 | 2.22 | 0 | 0 | 0 | 2.25 | 2.27 | 2.16 | 0 | 0 | 0 | 0 | 0 | 2.11 | 0 | 2.4 | 1.8 | 0 | 0 | 0 | 0 |
| 15SBCL1428STA | 0 | 0 | 0 | 0 | 0 | 2.3 | 0 | 2.4 | 0 | 0 | 0 | 2.7 | 2.4 | 2.5 | 0 | 0 | 0 | 0 | 0 | 2.2 | 0 | 2.1 | 1.5 | 0 | 0 | 0 | 0 |
| 15SBCL1430STA | 0 | 0 | 0 | 0 | 0 | 2.14 | 0 | 2.10 | 0 | 0 | 0 | 2.26 | 2.12 | 2.26 | 0 | 0 | 0 | 0 | 0 | 2.6 | 0 | 2.7 | 1.6 | 0 | 0 | 0 | 0 |
| 15SBCL1434STA | 0 | 0 | 0 | 0 | 0 | 0 | 0 | 0 | 0 | 0 | 0 | 0 | 0 | 0 | 0 | 0 | 0 | 0 | 0 | 0 | 0 | 13.1 | 0 | 1.4 | 0 | 0 | 0 |
| 15SBCL1438STA | 0 | 0 | 4.2 | 0 | 0 | 0 | 0 | 9.1 | 0 | 0 | 1.1 | 0 | 7.2 | 8.1 | 0 | 0 | 0 | 0 | 0 | 8.1 | 0 | 19.1 | 7.2 | 1.6 | 0 | 0 | 3.3 |
| 15SBCL1506STA | 2.18 | 0 | 0 | 0 | 0 | 0 | 0 | 0 | 0 | 5.12 | 0 | 0 | 0 | 0 | 0 | 4.13 | 0 | 0 | 0 | 0 | 0 | 7.22 | 0 | 0 | 0 | 0 | 0 |
| 15SBCL1507STA | 0 | 0 | 0 | 0 | 0 | 2.19 | 0 | 2.15 | 0 | 0 | 0 | 2.18 | 2.18 | 2.18 | 3.10 | 0 | 0 | 0 | 0 | 0 | 0 | 4.4 | 0 | 0 | 0 | 0 | 0 |
| 15SBCL1509STA | 0 | 0 | 0 | 0 | 0 | 0 | 0 | 0 | 0 | 0 | 0 | 0 | 0 | 0 | 2.5 | 0 | 0 | 0 | 0 | 0 | 0 | 1.3 | 0 | 0 | 0 | 0 | 0 |
| 15SBCL1517STA | 0 | 0 | 5.1 | 0 | 0 | 2.21 | 0 | 10.3 | 0 | 0 | 1.4 | 8.3 | 2.22 | 2.4 | 0 | 0 | 0 | 0 | 0 | 9.3 | 0 | 17.4 | 0 | 0 | 0 | 0 | 0 |
| 15SBCL1520STA | 2.21 | 0 | 0 | 0 | 0 | 0 | 2.8 | 0 | 0 | 0 | 0 | 0 | 0 | 0 | 0 | 0 | 0 | 0 | 0 | 0 | 0 | 6.1 | 0 | 0 | 0 | 0 | 0 |
| 15SBCL1527STA | 0 | 0 | 0 | 0 | 0 | 2.26 | 0 | 10.1 | 0 | 0 | 0 | 8.2 | 2.9 | 2.8 | 0 | 0 | 0 | 0 | 0 | 9.1 | 0 | 17.2 | 0 | 0 | 0 | 0 | 0 |
| 15SBCL1540STA | 2.12 | 0 | 0 | 0 | 0 | 0 | 2.5 | 0 | 0 | 0 | 0 | 0 | 0 | 0 | 0 | 0 | 0 | 0 | 0 | 0 | 0 | 6.10 | 0 | 0 | 0 | 0 | 0 |
| 15SBCL1548STA | 2.9 | 0 | 0 | 0 | 0 | 0 | 2.3 | 0 | 0 | 0 | 0 | 0 | 0 | 0 | 0 | 0 | 0 | 0 | 0 | 0 | 0 | 6.12 | 0 | 0 | 0 | 0 | 0 |
| 15SBCL1550STA | 0 | 0 | 0 | 0 | 0 | 2.11 | 0 | 5.1 | 0 | 0 | 0 | 2.8 | 2.20 | 2.7 | 3.3 | 0 | 0 | 0 | 0 | 0 | 0 | 4.13 | 0 | 0 | 0 | 0 | 0 |
| 15SBCL1560STA | 2.11 | 0 | 0 | 0 | 0 | 0 | 2.7 | 0 | 0 | 5.13 | 0 | 0 | 0 | 0 | 0 | 4.4 | 0 | 0 | 0 | 0 | 0 | 6.13 | 0 | 0 | 0 | 0 | 0 |
| 15SBCL1565STA | 2.3 | 0 | 0 | 0 | 0 | 0 | 2.3 | 0 | 0 | 5.14 | 0 | 0 | 0 | 0 | 0 | 4.14 | 0 | 0 | 0 | 0 | 0 | 6.5 | 0 | 0 | 0 | 0 | 0 |
| 15SBCL1570STA | 2.6 | 0 | 0 | 0 | 0 | 0 | 2.3 | 0 | 0 | 5.11 | 0 | 0 | 0 | 0 | 0 | 4.12 | 0 | 0 | 0 | 0 | 0 | 6.1 | 0 | 0 | 0 | 0 | 0 |
| 15SBCL1571STA | 2.16 | 0 | 0 | 0 | 0 | 0 | 2.9 | 0 | 0 | 5.2 | 0 | 0 | 0 | 0 | 0 | 4.3 | 0 | 0 | 0 | 0 | 0 | 6.6 | 0 | 0 | 0 | 0 | 0 |
| 15SBCL1576STA | 2.11 | 0 | 0 | 0 | 0 | 0 | 2.7 | 0 | 0 | 5.13 | 0 | 0 | 0 | 0 | 0 | 4.4 | 0 | 0 | 0 | 0 | 0 | 6.9 | 0 | 0 | 0 | 0 | 0 |
| 17SBCL03STA | 0 | 0 | 0 | 0 | 0 | 0 | 0 | 0 | 0 | 0 | 0 | 0 | 0 | 0 | 0 | 0 | 0 | 0 | 0 | 0 | 0 | 12.1 | 0 | 0 | 0 | 0 | 0 |
| 17SBCL07STA | 0 | 0 | 0 | 0 | 0 | 0 | 0 | 0 | 0 | 0 | 0 | 0 | 0 | 0 | 0 | 0 | 0 | 0 | 0 | 0 | 0 | 12.5 | 0 | 0 | 0 | 0 | 0 |
| 17SBCL08STA | 0 | 0 | 0 | 0 | 0 | 2.7 | 0 | 2.8 | 0 | 0 | 0 | 2.21 | 2.23 | 2.19 | 0 | 0 | 0 | 0 | 0 | 0 | 0 | 4.1 | 0 | 0 | 0 | 0 | 0 |
| 17SBCL09STA | 0 | 0 | 0 | 0 | 0 | 2.12 | 0 | 2.9 | 0 | 0 | 0 | 2.12 | 2.11 | 2.12 | 0 | 0 | 0 | 0 | 0 | 0 | 0 | 4.10 | 0 | 0 | 0 | 0 | 0 |
| 17SBCL13STA | 2.14 | 0 | 0 | 0 | 0 | 2.25 | 2.4 | 2.21 | 0 | 5.9 | 0 | 2.24 | 2.26 | 2.14 | 0 | 4.11 | 0 | 0 | 0 | 0 | 0 | 6.3 | 0 | 0 | 0 | 0 | 1.3 |
| 17SBCL18STA | 0 | 0 | 0 | 0 | 0 | 0 | 0 | 0 | 0 | 0 | 0 | 0 | 0 | 0 | 0 | 0 | 0 | 0 | 0 | 0 | 0 | 9.1 | 4.1 | 0 | 0 | 0 | 0 |
| 17SBCL202STA | 0 | 0 | 0 | 0 | 0 | 2.2 | 0 | 2.2 | 0 | 0 | 0 | 2.1 | 2.1 | 2.1 | 0 | 0 | 0 | 0 | 0 | 2.1 | 0 | 2.6 | 1.5 | 0 | 0 | 0 | 0 |
| 17SBCL208STA | 0 | 0 | 0 | 0 | 0 | 2.12 | 0 | 2.9 | 0 | 0 | 0 | 2.12 | 2.11 | 2.12 | 0 | 0 | 0 | 0 | 0 | 2.5 | 0 | 2.3 | 1.5 | 0 | 0 | 0 | 0 |
| 17SBCL214STA | 0 | 0 | 0 | 0 | 0 | 2.8 | 0 | 2.6 | 0 | 0 | 0 | 2.9 | 2.7 | 2.10 | 0 | 0 | 0 | 0 | 0 | 2.7 | 0 | 2.9 | 1.1 | 0 | 0 | 0 | 0 |
| 17SBCL220STA | 0 | 0 | 0 | 0 | 0 | 2.2 | 0 | 2.2 | 0 | 0 | 0 | 2.1 | 2.1 | 2.1 | 0 | 0 | 0 | 0 | 0 | 2.1 | 0 | 2.2 | 1.5 | 0 | 0 | 0 | 0 |
| 17SBCL223STA | 0 | 0 | 0 | 0 | 0 | 0 | 0 | 0 | 0 | 0 | 0 | 0 | 0 | 0 | 0 | 0 | 0 | 0 | 0 | 0 | 0 | 12.6 | 0 | 0 | 0 | 0 | 0 |
| 17SBCL225STA | 0 | 0 | 0 | 0 | 0 | 2.1 | 0 | 2.19 | 0 | 0 | 0 | 2.16 | 2.2 | 2.6 | 0 | 0 | 0 | 0 | 0 | 2.12 | 0 | 2.9 | 1.2 | 0 | 0 | 0 | 0 |
| 17SBCL532STA | 0 | 0 | 0 | 0 | 0 | 0 | 2.7 | 0 | 0 | 0 | 0 | 0 | 0 | 0 | 0 | 0 | 0 | 0 | 0 | 0 | 0 | 6.13 | 0 | 0 | 0 | 0 | 0 |
| 17SBCL533STA | 0 | 0 | 0 | 0 | 0 | 0 | 2.7 | 0 | 0 | 0 | 0 | 0 | 0 | 0 | 0 | 0 | 0 | 0 | 0 | 0 | 0 | 6.13 | 0 | 0 | 0 | 0 | 0 |
| 17SBCL580STA | 2.16 | 0 | 0 | 2.1 | 0 | 2.9 | 0 | 2.3 | 1.8 | 0 | 0 | 2.5 | 2.8 | 2.21 | 0 | 0 | 2.1 | 0 | 0 | 0 | 0 | 4.13 | 0 | 0 | 0 | 0 | 4.7 |
| 17SBCL585STA | 2.16 | 0 | 0 | 2.1 | 0 | 2.9 | 0 | 2.3 | 1.8 | 0 | 0 | 2.5 | 2.8 | 2.21 | 0 | 0 | 2.1 | 0 | 0 | 0 | 0 | 4.7 | 0 | 0 | 0 | 0 | 4.6 |
| 17SBCL586STA | 2.13 | 0 | 0 | 2.1 | 0 | 2.4 | 0 | 2.16 | 1.8 | 0 | 0 | 2.19 | 2.6 | 2.20 | 0 | 0 | 2.1 | 0 | 0 | 0 | 0 | 4.12 | 0 | 0 | 0 | 0 | 4.1 |
| 17SBCL778STA | 0 | 0 | 0 | 0 | 0 | 0 | 0 | 0 | 0 | 0 | 0 | 0 | 0 | 0 | 0 | 0 | 0 | 0 | 0 | 0 | 0 | 12.2 | 0 | 0 | 0 | 0 | 0 |
| 1A | 0 | 0 | 0 | 0 | 0 | 2.21 | 0 | 2.20 | 0 | 0 | 0 | 2.4 | 2.22 | 2.4 | 3.4 | 0 | 0 | 0 | 0 | 0 | 0 | 4.7 | 0 | 0 | 0 | 0 | 0 |
| 294E | 2.4 | 0 | 0 | 1.3 | 0 | 0 | 0 | 0 | 1.4 | 0 | 0 | 0 | 0 | 0 | 0 | 0 | 1.8 | 0 | 0 | 0 | 0 | 7.11 | 0 | 0 | 0 | 0 | 0 |
| 333E | 0 | 0 | 0 | 0 | 0 | 0 | 0 | 0 | 0 | 0 | 0 | 0 | 0 | 0 | 0 | 0 | 0 | 0 | 0 | 0 | 0 | 12.9 | 0 | 0 | 0 | 0 | 0 |
| 367F | 0 | 0 | 0 | 0 | 0 | 2.19 | 0 | 2.15 | 0 | 0 | 0 | 2.18 | 2.18 | 2.18 | 3.2 | 0 | 0 | 0 | 0 | 0 | 0 | 4.9 | 0 | 0 | 0 | 0 | 0 |
| 36A | 0 | 0 | 0 | 0 | 0 | 0 | 2.6 | 0 | 0 | 0 | 0 | 0 | 0 | 0 | 0 | 0 | 0 | 0 | 0 | 0 | 0 | 6.6 | 0 | 0 | 0 | 0 | 0 |
| 388F | 2.22 | 0 | 0 | 0 | 0 | 0 | 0 | 0 | 0 | 0 | 0 | 0 | 0 | 0 | 0 | 0 | 0 | 0 | 0 | 0 | 0 | 7.10 | 0 | 0 | 0 | 0 | 0 |
| 42A | 1.1 | 1.2 | 0 | 0 | 0 | 0 | 0 | 0 | 0 | 1.1 | 0 | 0 | 0 | 0 | 0 | 1.2 | 0 | 0 | 0 | 0 | 0 | 7.8 | 0 | 0 | 0 | 0 | 0 |
| 43A | 0 | 0 | 1.1 | 0 | 0 | 1.2 | 1.4 | 1.2 | 0 | 0 | 1.2 | 1.2 | 1.2 | 1.2 | 0 | 0 | 0 | 0 | 0 | 5.2 | 0 | 10.2 | 0 | 0 | 0 | 0 | 0 |
| 44A | 0 | 0 | 6.2 | 0 | 0 | 0 | 0 | 0 | 0 | 7.1 | 0 | 0 | 0 | 0 | 6.1 | 1.1 | 0 | 0 | 0 | 0 | 0 | 7.1 | 0 | 3.2 | 0 | 0 | 0 |
| 45A | 0 | 0 | 0 | 3.1 | 0 | 0 | 0 | 0 | 1.4 | 0 | 0 | 0 | 0 | 0 | 0 | 0 | 1.8 | 0 | 0 | 0 | 0 | 7.21 | 0 | 0 | 0 | 0 | 0 |
| 46A | 0 | 0 | 0 | 0 | 1.1 | 0 | 0 | 0 | 0 | 0 | 0 | 0 | 0 | 0 | 0 | 7.1 | 0 | 0 | 0 | 0 | 0 | 0 | 0 | 4.1 | 0 | 0 | 0 |
| 47A | 0 | 0 | 0 | 0 | 0 | 0 | 0 | 0 | 0 | 6.2 | 0 | 0 | 0 | 0 | 0 | 6.2 | 0 | 0 | 0 | 0 | 0 | 7.24 | 0 | 0 | 0 | 0 | 1.6 |
| GCA_000009005 | 0 | 0 | 4.4 | 0 | 0 | 0 | 0 | 8.2 | 0 | 0 | 1.9 | 0 | 7.3 | 8.3 | 0 | 0 | 0 | 0 | 0 | 8.4 | 0 | 14.5 | 7.3 | 1.3 | 0 | 0 | 3.4 |
| GCA_000010465 | 1.3 | 0 | 0 | 0 | 0 | 0 | 0 | 0 | 0 | 0 | 0 | 0 | 0 | 0 | 0 | 0 | 0 | 0 | 0 | 0 | 0 | 7.17 | 0 | 0 | 0 | 0 | 0 |
| GCA_000011265 | 2.10 | 0 | 10.1 | 0 | 0 | 0 | 2.3 | 0 | 0 | 5.7 | 8.1 | 0 | 0 | 0 | 0 | 4.8 | 0 | 0 | 0 | 0 | 0 | 6.6 | 0 | 0 | 0 | 0 | 0 |
| GCA_000011505 | 1.3 | 0 | 0 | 0 | 0 | 3.11 | 0 | 3.12 | 0 | 0 | 0 | 3.11 | 3.12 | 3.11 | 0 | 0 | 0 | 0 | 0 | 1.12 | 0 | 0 | 0 | 0 | 0 | 0 | 0 |
| GCA_000013425 | 0 | 0 | 0 | 0 | 0 | 0 | 0 | 0 | 0 | 0 | 0 | 0 | 0 | 0 | 0 | 0 | 0 | 0 | 0 | 0 | 0 | 7.17 | 0 | 0 | 0 | 0 | 0 |
| GCA_000017085 | 0 | 0 | 0 | 0 | 0 | 0 | 0 | 0 | 0 | 3.1 | 0 | 0 | 0 | 0 | 0 | 2.1 | 0 | 0 | 0 | 0 | 0 | 7.17 | 0 | 0 | 0 | 0 | 0 |
| GCA_000025145 | 0 | 0 | 0 | 0 | 0 | 9.1 | 0 | 2.17 | 0 | 0 | 0 | 2.20 | 2.25 | 2.25 | 3.7 | 0 | 0 | 0 | 0 | 0 | 0 | 4.6 | 0 | 0 | 0 | 0 | 0 |
| GCA_000027045 | 2.10 | 0 | 0 | 0 | 0 | 0 | 0 | 0 | 0 | 6.1 | 0 | 0 | 0 | 0 | 0 | 6.1 | 0 | 0 | 0 | 0 | 0 | 7.17 | 0 | 0 | 0 | 0 | 0 |
| GCA_000144955 | 0 | 0 | 0 | 0 | 0 | 0 | 0 | 0 | 0 | 0 | 0 | 0 | 0 | 0 | 0 | 0 | 0 | 0 | 0 | 0 | 0 | 27.1 | 0 | 5.1 | 0 | 0 | 0 |
| GCA_000153665 | 0 | 0 | 0 | 0 | 0 | 0 | 0 | 0 | 0 | 0 | 0 | 0 | 0 | 0 | 2.1 | 0 | 0 | 0 | 0 | 0 | 0 | 1.4 | 0 | 0 | 0 | 0 | 0 |
| GCA_000204665 | 2.10 | 0 | 0 | 0 | 0 | 0 | 0 | 0 | 0 | 6.1 | 0 | 0 | 0 | 0 | 0 | 5.1 | 0 | 0 | 0 | 0 | 0 | 7.17 | 0 | 0 | 0 | 0 | 0 |
| GCA_000210315 | 3.1 | 0 | 2.2 | 0 | 0 | 0 | 0 | 0 | 0 | 0 | 2.2 | 0 | 0 | 0 | 0 | 0 | 0 | 0 | 0 | 0 | 0 | 29.1 | 0 | 0 | 0 | 0 | 2.7 |
| GCA_000237125 | 0 | 1.7 | 0 | 0 | 0 | 0 | 0 | 0 | 0 | 4.1 | 0 | 0 | 0 | 0 | 0 | 3.1 | 0 | 0 | 0 | 0 | 0 | 9.2 | 4.2 | 0 | 0 | 0 | 0 |
| GCA_000237265 | 0 | 0 | 0 | 0 | 0 | 0 | 0 | 0 | 0 | 0 | 0 | 0 | 0 | 0 | 0 | 0 | 0 | 0 | 0 | 0 | 0 | 14.2 | 0 | 0 | 0 | 0 | 0 |
| GCA_000239235 | 0 | 0 | 0 | 0 | 0 | 0 | 0 | 0 | 0 | 0 | 0 | 0 | 0 | 0 | 0 | 0 | 0 | 0 | 0 | 0 | 0 | 30.1 | 9.1 | 0 | 0 | 0 | 0 |
| GCA_000245495 | 0 | 0 | 0 | 0 | 0 | 0 | 0 | 0 | 0 | 0 | 0 | 0 | 0 | 0 | 0 | 0 | 0 | 0 | 0 | 0 | 0 | 7.17 | 0 | 0 | 0 | 0 | 0 |
| GCA_000262835 | 0 | 0 | 0 | 0 | 0 | 2.22 | 0 | 2.17 | 0 | 0 | 0 | 2.20 | 2.25 | 2.25 | 0 | 0 | 0 | 0 | 0 | 0 | 0 | 4.6 | 0 | 0 | 0 | 0 | 0 |
| GCA_000296595 | 0 | 0 | 0 | 0 | 0 | 0 | 0 | 0 | 0 | 0 | 0 | 0 | 0 | 0 | 0 | 0 | 0 | 0 | 0 | 0 | 0 | 0 | 0 | 0 | 0 | 0 | 0 |
| GCA_000336295 | 4.1 | 0 | 6.4 | 0 | 0 | 0 | 0 | 6.3 | 0 | 0 | 0 | 10.1 | 2.25 | 2.25 | 0 | 0 | 0 | 0 | 0 | 7.5 | 0 | 23.1 | 0 | 3.1 | 1.1 | 1.1 | 0 |
| GCA_000362085 | 0 | 0 | 0 | 2.1 | 0 | 2.22 | 0 | 2.17 | 0 | 0 | 0 | 2.20 | 2.25 | 2.25 | 0 | 0 | 1.8 | 0 | 0 | 0 | 0 | 4.8 | 0 | 0 | 0 | 0 | 0 |
| GCA_000382965 | 2.10 | 0 | 0 | 0 | 0 | 2.22 | 0 | 2.17 | 0 | 0 | 0 | 2.20 | 2.25 | 2.25 | 0 | 0 | 0 | 0 | 0 | 0 | 0 | 4.6 | 0 | 0 | 0 | 0 | 0 |
| GCA_000412775 | 0 | 0 | 0 | 0 | 0 | 2.22 | 0 | 6.3 | 0 | 0 | 0 | 5.3 | 0 | 5.1 | 0 | 0 | 0 | 0 | 0 | 4.1 | 0 | 0 | 0 | 0 | 0 | 0 | 0 |
| GCA_000418345 | 0 | 0 | 0 | 0 | 0 | 0 | 0 | 0 | 0 | 0 | 0 | 0 | 0 | 0 | 0 | 0 | 0 | 0 | 0 | 0 | 0 | 7.17 | 0 | 0 | 0 | 0 | 0 |
| GCA_000452385 | 0 | 0 | 0 | 0 | 0 | 0 | 0 | 0 | 0 | 0 | 0 | 0 | 0 | 0 | 5.1 | 0 | 0 | 0 | 0 | 0 | 0 | 22.1 | 0 | 0 | 0 | 0 | 0 |
| GCA_000463055 | 0 | 0 | 0 | 0 | 0 | 7.3 | 0 | 2.13 | 0 | 0 | 0 | 2.6 | 6.2 | 7.2 | 0 | 0 | 0 | 0 | 0 | 7.3 | 0 | 6.6 | 0 | 0 | 0 | 0 | 0 |
| GCA_000512505 | 5.1 | 0 | 0 | 0 | 0 | 0 | 2.3 | 0 | 0 | 0 | 0 | 0 | 0 | 0 | 0 | 0 | 0 | 0 | 0 | 0 | 0 | 0 | 0 | 0 | 0 | 0 | 0 |
| GCA_000551805 | 2.10 | 1.7 | 0 | 0 | 0 | 0 | 0 | 0 | 0 | 1.6 | 0 | 0 | 0 | 0 | 0 | 1.4 | 0 | 0 | 0 | 0 | 0 | 7.17 | 0 | 0 | 0 | 0 | 0 |
| GCA_000555645 | 0 | 0 | 0 | 0 | 0 | 2.22 | 0 | 2.17 | 0 | 0 | 0 | 2.20 | 2.25 | 2.25 | 0 | 0 | 0 | 0 | 0 | 0 | 0 | 4.6 | 0 | 0 | 0 | 0 | 0 |
| GCA_000564485 | 0 | 1.7 | 0 | 0 | 0 | 0 | 0 | 0 | 0 | 4.1 | 0 | 0 | 0 | 0 | 0 | 1.4 | 0 | 0 | 0 | 0 | 0 | 9.2 | 4.2 | 0 | 0 | 0 | 0 |
| GCA_000568455 | 0 | 0 | 0 | 0 | 0 | 0 | 0 | 0 | 0 | 3.1 | 0 | 0 | 0 | 0 | 0 | 2.1 | 0 | 0 | 0 | 0 | 0 | 7.17 | 0 | 0 | 0 | 0 | 0 |
| GCA_000577925 | 1.3 | 0 | 0 | 0 | 0 | 3.11 | 0 | 3.12 | 0 | 2.1 | 0 | 0 | 3.12 | 3.11 | 0 | 2.1 | 0 | 0 | 0 | 1.12 | 0 | 0 | 0 | 0 | 0 | 0 | 0 |
| GCA_000586795 | 0 | 0 | 0 | 0 | 0 | 2.22 | 0 | 6.3 | 2.1 | 0 | 0 | 5.3 | 2.25 | 2.25 | 0 | 0 | 4.1 | 3.1 | 1.1 | 7.5 | 0 | 0 | 0 | 0 | 0 | 0 | 0 |
| GCA_000595185 | 0 | 0 | 0 | 0 | 0 | 2.22 | 0 | 2.17 | 0 | 0 | 0 | 0 | 2.25 | 2.25 | 0 | 0 | 0 | 0 | 0 | 0 | 0 | 4.6 | 0 | 0 | 0 | 0 | 0 |
| GCA_000636155 | 0 | 0 | 0 | 0 | 0 | 7.1 | 0 | 2.17 | 0 | 0 | 0 | 2.20 | 6.3 | 7.4 | 0 | 0 | 0 | 0 | 0 | 7.1 | 0 | 15.1 | 6.1 | 0 | 2.1 | 1.1 | 0 |
| GCA_000637195 | 0 | 0 | 0 | 0 | 0 | 0 | 0 | 0 | 0 | 0 | 0 | 0 | 0 | 0 | 0 | 0 | 0 | 0 | 0 | 0 | 0 | 12.6 | 0 | 0 | 0 | 0 | 0 |
| GCA_000638335 | 0 | 0 | 0 | 0 | 0 | 0 | 0 | 0 | 0 | 0 | 0 | 0 | 0 | 0 | 0 | 0 | 0 | 0 | 0 | 0 | 0 | 0 | 0 | 0 | 0 | 0 | 0 |
| GCA_000695215 | 0 | 0 | 5.3 | 0 | 0 | 2.22 | 0 | 10.2 | 0 | 0 | 1.5 | 8.1 | 2.25 | 2.25 | 0 | 0 | 0 | 0 | 0 | 9.4 | 0 | 17.1 | 0 | 0 | 0 | 0 | 0 |
| GCA_000731515 | 0 | 0 | 0 | 0 | 0 | 2.22 | 0 | 2.17 | 0 | 0 | 0 | 2.20 | 2.25 | 9.1 | 4.1 | 0 | 0 | 0 | 0 | 0 | 0 | 4.6 | 0 | 0 | 0 | 0 | 0 |
| GCA_000735755 | 0 | 0 | 0 | 0 | 0 | 2.16 | 0 | 0 | 0 | 0 | 0 | 5.1 | 2.13 | 2.2 | 0 | 0 | 0 | 0 | 0 | 11.1 | 0 | 7.25 | 0 | 0 | 0 | 0 | 0 |
| GCA_000736455 | 0 | 0 | 0 | 0 | 0 | 0 | 0 | 0 | 0 | 0 | 0 | 0 | 0 | 0 | 0 | 0 | 0 | 0 | 0 | 0 | 0 | 7.17 | 0 | 0 | 0 | 0 | 0 |
| GCA_000756205 | 0 | 0 | 0 | 0 | 0 | 3.11 | 0 | 3.12 | 0 | 0 | 0 | 3.11 | 3.12 | 3.11 | 0 | 0 | 0 | 0 | 0 | 1.12 | 0 | 0 | 0 | 0 | 0 | 0 | 0 |
| GCA_000772025 | 4.1 | 0 | 0 | 0 | 0 | 3.11 | 0 | 3.12 | 0 | 0 | 0 | 3.11 | 8.1 | 3.11 | 0 | 0 | 0 | 0 | 0 | 1.12 | 0 | 0 | 0 | 0 | 0 | 0 | 4.3 |
| GCA_000828035 | 4.1 | 0 | 6.4 | 0 | 0 | 0 | 0 | 6.3 | 0 | 0 | 0 | 10.1 | 2.25 | 2.25 | 0 | 0 | 0 | 0 | 0 | 7.5 | 0 | 23.1 | 0 | 3.1 | 1.1 | 1.1 | 0 |
| GCA_000878105 | 0 | 0 | 0 | 0 | 3.1 | 0 | 0 | 0 | 0 | 0 | 0 | 0 | 0 | 0 | 0 | 0 | 0 | 0 | 0 | 0 | 0 | 31.1 | 0 | 0 | 0 | 0 | 0 |
| GCA_000934285 | 0 | 0 | 0 | 0 | 0 | 0 | 0 | 0 | 0 | 0 | 0 | 0 | 0 | 0 | 0 | 0 | 0 | 0 | 0 | 0 | 0 | 0 | 6.2 | 0 | 0 | 0 | 0 |
| GCA_001018955 | 0 | 0 | 0 | 0 | 0 | 0 | 0 | 0 | 0 | 0 | 0 | 0 | 0 | 0 | 0 | 0 | 0 | 0 | 0 | 0 | 0 | 7.17 | 0 | 0 | 0 | 0 | 0 |
| GCA_001046335 | 0 | 0 | 0 | 0 | 0 | 0 | 0 | 0 | 0 | 3.1 | 0 | 0 | 0 | 0 | 0 | 2.1 | 0 | 0 | 0 | 0 | 0 | 7.17 | 0 | 0 | 0 | 0 | 0 |
| GCA_001049655 | 0 | 0 | 0 | 0 | 0 | 0 | 0 | 0 | 0 | 0 | 0 | 0 | 0 | 0 | 0 | 0 | 0 | 0 | 0 | 0 | 0 | 7.17 | 0 | 0 | 0 | 0 | 0 |
| GCA_001060255 | 2.10 | 0 | 0 | 0 | 0 | 0 | 0 | 0 | 0 | 0 | 0 | 0 | 0 | 0 | 0 | 0 | 0 | 0 | 0 | 0 | 0 | 7.12 | 0 | 0 | 0 | 0 | 0 |
| GCA_001062005 | 0 | 0 | 0 | 0 | 0 | 2.22 | 0 | 2.17 | 0 | 0 | 0 | 2.20 | 2.25 | 2.25 | 0 | 0 | 0 | 0 | 0 | 2.10 | 0 | 18.1 | 0 | 0 | 0 | 0 | 0 |
| GCA_001065475 | 0 | 0 | 0 | 0 | 0 | 0 | 0 | 0 | 0 | 0 | 0 | 0 | 0 | 0 | 0 | 0 | 0 | 0 | 0 | 0 | 0 | 24.1 | 0 | 0 | 0 | 0 | 0 |
| GCA_001184325 | 4.1 | 0 | 0 | 0 | 0 | 0 | 4.1 | 0 | 0 | 0 | 0 | 0 | 0 | 0 | 0 | 0 | 0 | 0 | 0 | 0 | 0 | 6.6 | 0 | 0 | 0 | 0 | 0 |
| GCA_001200275 | 0 | 0 | 0 | 0 | 0 | 0 | 0 | 0 | 0 | 0 | 0 | 0 | 0 | 0 | 0 | 0 | 0 | 0 | 0 | 0 | 0 | 7.17 | 0 | 0 | 0 | 0 | 0 |
| GCA_001208785 | 0 | 0 | 5.3 | 0 | 0 | 2.22 | 0 | 0 | 0 | 0 | 1.5 | 8.1 | 2.25 | 2.25 | 0 | 0 | 0 | 0 | 0 | 9.4 | 0 | 17.1 | 0 | 0 | 0 | 0 | 0 |
| GCA_001209085 | 0 | 0 | 0 | 0 | 0 | 0 | 0 | 0 | 0 | 0 | 0 | 0 | 0 | 0 | 0 | 0 | 0 | 0 | 0 | 0 | 0 | 5.1 | 0 | 0 | 0 | 0 | 0 |
| GCA_001211365 | 0 | 0 | 0 | 0 | 0 | 2.22 | 0 | 10.2 | 0 | 0 | 0 | 8.1 | 2.25 | 2.25 | 0 | 0 | 0 | 0 | 0 | 9.4 | 0 | 17.1 | 0 | 0 | 0 | 0 | 0 |
| GCA_001225625 | 0 | 0 | 0 | 0 | 0 | 2.6 | 0 | 10.2 | 0 | 0 | 0 | 8.1 | 2.25 | 2.25 | 0 | 0 | 0 | 0 | 0 | 9.4 | 0 | 17.1 | 0 | 0 | 0 | 0 | 0 |
| GCA_001228145 | 0 | 0 | 0 | 0 | 0 | 0 | 0 | 0 | 0 | 0 | 0 | 8.5 | 0 | 0 | 0 | 0 | 0 | 0 | 0 | 0 | 0 | 0 | 0 | 0 | 0 | 0 | 0 |
| GCA_001291025 | 2.10 | 1.10 | 0 | 0 | 0 | 0 | 0 | 0 | 0 | 1.3 | 0 | 0 | 0 | 0 | 0 | 8.1 | 0 | 0 | 0 | 0 | 0 | 7.17 | 0 | 0 | 0 | 0 | 0 |
| GCA_001297475 | 0 | 2.1 | 0 | 0 | 0 | 4.2 | 0 | 4.2 | 0 | 3.1 | 0 | 4.2 | 4.2 | 4.2 | 0 | 2.1 | 0 | 0 | 0 | 3.2 | 0 | 5.1 | 2.1 | 2.1 | 0 | 0 | 0 |
| GCA_001349875 | 0 | 0 | 5.3 | 0 | 0 | 2.22 | 0 | 10.2 | 0 | 0 | 1.5 | 8.1 | 2.25 | 2.25 | 0 | 0 | 0 | 0 | 0 | 9.4 | 0 | 17.1 | 0 | 0 | 0 | 0 | 0 |
| GCA_001350955 | 0 | 1.9 | 0 | 0 | 0 | 0 | 0 | 0 | 0 | 1.2 | 0 | 0 | 0 | 0 | 0 | 1.1 | 0 | 0 | 0 | 0 | 0 | 7.17 | 0 | 0 | 0 | 0 | 0 |
| GCA_001353795 | 0 | 0 | 0 | 0 | 0 | 0 | 0 | 0 | 0 | 3.1 | 0 | 0 | 0 | 0 | 0 | 2.1 | 0 | 0 | 0 | 0 | 0 | 7.17 | 0 | 0 | 0 | 0 | 0 |
| GCA_001411695 | 0 | 0 | 0 | 0 | 0 | 3.11 | 0 | 3.12 | 0 | 0 | 0 | 3.11 | 3.12 | 3.11 | 0 | 0 | 0 | 0 | 0 | 1.12 | 0 | 0 | 0 | 0 | 0 | 0 | 0 |
| GCA_001411705 | 0 | 0 | 0 | 0 | 0 | 2.22 | 0 | 2.17 | 3.1 | 0 | 0 | 11.1 | 2.25 | 2.25 | 3.12 | 0 | 1.5 | 2.1 | 0 | 0 | 0 | 0 | 0 | 0 | 0 | 0 | 0 |
| GCA_001456215 | 0 | 1.7 | 0 | 0 | 0 | 0 | 0 | 0 | 0 | 4.1 | 0 | 0 | 0 | 0 | 0 | 9.1 | 0 | 0 | 0 | 0 | 0 | 9.2 | 4.2 | 0 | 0 | 0 | 0 |
| GCA_001679585 | 1.3 | 0 | 0 | 0 | 0 | 0 | 0 | 0 | 0 | 0 | 0 | 0 | 0 | 0 | 0 | 0 | 0 | 0 | 0 | 0 | 0 | 7.17 | 0 | 0 | 0 | 0 | 0 |
| GCA_001691505 | 0 | 0 | 0 | 0 | 0 | 2.22 | 0 | 2.17 | 0 | 0 | 0 | 2.20 | 2.25 | 2.25 | 0 | 0 | 0 | 0 | 0 | 2.10 | 0 | 18.1 | 0 | 0 | 0 | 0 | 0 |
| GCA_001887075 | 0 | 0 | 0 | 0 | 0 | 0 | 0 | 0 | 0 | 0 | 0 | 0 | 0 | 0 | 0 | 0 | 0 | 0 | 0 | 0 | 0 | 0 | 0 | 0 | 0 | 0 | 0 |
| GCA_001900185 | 0 | 0 | 0 | 0 | 0 | 0 | 0 | 0 | 0 | 0 | 0 | 0 | 0 | 0 | 0 | 0 | 0 | 0 | 0 | 0 | 0 | 7.17 | 0 | 0 | 0 | 0 | 0 |
| GCA_001936705 | 0 | 0 | 0 | 2.1 | 0 | 2.22 | 0 | 2.17 | 1.8 | 0 | 0 | 2.20 | 2.25 | 2.25 | 0 | 0 | 1.8 | 0 | 0 | 0 | 0 | 4.6 | 0 | 0 | 0 | 0 | 0 |
| GCA_001936775 | 0 | 0 | 0 | 2.1 | 0 | 0 | 0 | 0 | 0 | 0 | 0 | 0 | 0 | 0 | 0 | 0 | 1.8 | 0 | 0 | 0 | 0 | 4.6 | 0 | 0 | 0 | 0 | 0 |
| GCA_001936855 | 0 | 0 | 0 | 0 | 0 | 0 | 0 | 0 | 0 | 0 | 0 | 0 | 0 | 0 | 3.15 | 0 | 0 | 0 | 0 | 0 | 0 | 4.6 | 0 | 0 | 0 | 0 | 0 |
| GCA_001936895 | 0 | 0 | 0 | 0 | 0 | 0 | 0 | 0 | 0 | 0 | 0 | 0 | 0 | 0 | 3.7 | 0 | 0 | 0 | 0 | 0 | 0 | 4.6 | 0 | 0 | 0 | 0 | 0 |
| GCA_001936915 | 0 | 0 | 0 | 0 | 0 | 2.22 | 0 | 2.17 | 0 | 0 | 0 | 2.20 | 2.25 | 2.25 | 0 | 0 | 0 | 0 | 0 | 0 | 0 | 4.6 | 0 | 0 | 0 | 0 | 0 |
| GCA_001936985 | 0 | 0 | 0 | 0 | 0 | 0 | 0 | 0 | 0 | 0 | 0 | 0 | 0 | 0 | 0 | 0 | 0 | 0 | 0 | 0 | 0 | 4.6 | 0 | 0 | 0 | 0 | 0 |
| GCA_001937005 | 0 | 0 | 0 | 4.1 | 0 | 0 | 0 | 0 | 1.8 | 0 | 0 | 2.20 | 0 | 2.12 | 0 | 0 | 1.8 | 0 | 0 | 0 | 0 | 4.6 | 0 | 0 | 0 | 0 | 0 |
| GCA_001996505 | 0 | 0 | 0 | 0 | 0 | 0 | 0 | 0 | 0 | 0 | 0 | 0 | 0 | 0 | 0 | 0 | 0 | 0 | 0 | 0 | 0 | 7.17 | 0 | 0 | 0 | 0 | 0 |
| GCA_002097325 | 2.10 | 0 | 0 | 0 | 0 | 0 | 0 | 0 | 0 | 1.5 | 0 | 0 | 0 | 0 | 0 | 1.4 | 0 | 0 | 0 | 0 | 0 | 7.17 | 0 | 0 | 0 | 0 | 0 |
| GCA_002097375 | 0 | 0 | 5.3 | 0 | 0 | 0 | 0 | 0 | 0 | 0 | 0 | 0 | 0 | 0 | 0 | 0 | 0 | 0 | 0 | 0 | 0 | 17.1 | 0 | 0 | 0 | 0 | 0 |
| GCA_002097415 | 0 | 0 | 0 | 0 | 0 | 0 | 0 | 0 | 0 | 0 | 0 | 0 | 0 | 0 | 0 | 0 | 0 | 0 | 0 | 0 | 0 | 17.1 | 0 | 0 | 0 | 0 | 0 |
| GCA_002097425 | 2.10 | 0 | 0 | 0 | 0 | 0 | 0 | 0 | 0 | 1.6 | 0 | 0 | 0 | 0 | 0 | 1.4 | 0 | 0 | 0 | 0 | 0 | 0 | 0 | 0 | 0 | 0 | 0 |
| GCA_002097625 | 2.10 | 0 | 0 | 0 | 0 | 0 | 0 | 0 | 0 | 1.6 | 0 | 0 | 0 | 0 | 0 | 1.4 | 0 | 0 | 0 | 0 | 0 | 0 | 0 | 0 | 0 | 0 | 0 |
| GCA_900017565 | 0 | 0 | 0 | 0 | 0 | 0 | 0 | 0 | 0 | 0 | 0 | 0 | 0 | 0 | 0 | 0 | 0 | 0 | 0 | 0 | 0 | 26.1 | 0 | 0 | 0 | 0 | 0 |
| GCA_900040355 | 2.10 | 0 | 0 | 0 | 0 | 0 | 2.3 | 0 | 0 | 5.7 | 0 | 0 | 0 | 0 | 0 | 4.8 | 0 | 0 | 0 | 0 | 0 | 6.6 | 0 | 0 | 0 | 0 | 0 |
| GCA_900043625 | 2.10 | 0 | 0 | 0 | 0 | 0 | 0 | 0 | 0 | 6.1 | 0 | 0 | 0 | 0 | 0 | 6.1 | 0 | 0 | 0 | 0 | 0 | 7.17 | 0 | 0 | 0 | 0 | 0 |
| GCA_900045235 | 0 | 0 | 0 | 0 | 0 | 0 | 0 | 0 | 0 | 0 | 0 | 0 | 0 | 0 | 0 | 0 | 0 | 0 | 0 | 0 | 0 | 17.1 | 0 | 0 | 0 | 0 | 0 |
| GCA_900045535 | 2.10 | 0 | 0 | 0 | 0 | 0 | 0 | 0 | 0 | 0 | 0 | 0 | 0 | 0 | 0 | 0 | 0 | 0 | 0 | 0 | 0 | 7.17 | 0 | 0 | 0 | 0 | 0 |
| GCA_900046425 | 0 | 0 | 0 | 0 | 0 | 2.22 | 0 | 2.17 | 0 | 0 | 0 | 2.20 | 6.3 | 7.1 | 0 | 0 | 0 | 0 | 0 | 7.1 | 0 | 16.1 | 0 | 0 | 0 | 0 | 0 |
| GCA_900070235 | 2.10 | 0 | 0 | 0 | 0 | 0 | 0 | 0 | 0 | 0 | 0 | 0 | 0 | 0 | 0 | 0 | 0 | 0 | 0 | 0 | 0 | 0 | 0 | 0 | 0 | 0 | 0 |
| GCA_900080815 | 0 | 0 | 0 | 0 | 0 | 2.23 | 0 | 2.12 | 0 | 0 | 0 | 2.2 | 2.17 | 0 | 0 | 0 | 0 | 0 | 0 | 0 | 0 | 4.5 | 0 | 0 | 0 | 0 | 0 |
| GCA_900097435 | 0 | 0 | 0 | 0 | 0 | 0 | 0 | 0 | 0 | 0 | 0 | 0 | 0 | 0 | 0 | 0 | 0 | 0 | 0 | 0 | 0 | 28.1 | 0 | 0 | 0 | 0 | 0 |
| GCA_900097485 | 0 | 2.1 | 0 | 0 | 0 | 4.2 | 0 | 4.2 | 0 | 0 | 0 | 4.2 | 4.2 | 4.2 | 0 | 0 | 0 | 0 | 0 | 3.2 | 0 | 5.1 | 3.1 | 2.1 | 0 | 0 | 0 |
| GCA_900097495 | 0 | 0 | 0 | 0 | 0 | 0 | 0 | 0 | 0 | 0 | 0 | 0 | 0 | 0 | 0 | 0 | 0 | 0 | 0 | 0 | 0 | 7.14 | 0 | 0 | 0 | 0 | 0 |
| GCA_900098155 | 0 | 0 | 0 | 0 | 0 | 0 | 0 | 0 | 0 | 0 | 0 | 0 | 0 | 0 | 0 | 0 | 0 | 0 | 0 | 0 | 0 | 12.6 | 0 | 1.4 | 0 | 0 | 0 |
| GCA_900098605 | 0 | 0 | 0 | 0 | 0 | 0 | 0 | 0 | 0 | 0 | 0 | 0 | 0 | 0 | 0 | 0 | 0 | 0 | 0 | 0 | 0 | 0 | 0 | 0 | 0 | 0 | 0 |
| GCA_900124965 | 0 | 0 | 0 | 0 | 0 | 0 | 0 | 0 | 0 | 0 | 0 | 0 | 0 | 0 | 0 | 0 | 0 | 0 | 0 | 0 | 0 | 0 | 0 | 0 | 0 | 0 | 0 |
| GCA_900125555 | 0 | 0 | 0 | 0 | 0 | 0 | 0 | 0 | 0 | 0 | 0 | 0 | 0 | 0 | 0 | 0 | 0 | 0 | 0 | 0 | 0 | 25.1 | 0 | 0 | 0 | 0 | 0 |
| GCA_900125885 | 0 | 0 | 0 | 0 | 0 | 0 | 0 | 0 | 0 | 0 | 0 | 0 | 0 | 0 | 0 | 0 | 0 | 0 | 0 | 0 | 0 | 20.1 | 0 | 0 | 0 | 0 | 0 |
| GCA_900126215 | 0 | 0 | 0 | 0 | 0 | 5.1 | 3.1 | 7.1 | 0 | 0 | 0 | 6.1 | 5.1 | 6.1 | 0 | 0 | 0 | 0 | 0 | 6.1 | 0 | 11.1 | 5.1 | 0 | 0 | 0 | 0 |
| GCA_900127985 | 0 | 2.1 | 0 | 0 | 0 | 8.1 | 0 | 4.2 | 0 | 0 | 0 | 9.1 | 4.2 | 4.2 | 0 | 0 | 0 | 0 | 0 | 10.1 | 0 | 5.1 | 8.1 | 2.1 | 0 | 0 | 0 |

Table SIV: Table of results obtained by genomic (NAuRA profiles), molecular biology (PCR profiles) and SE detection in food. The “Discordance” column highlights differential results by at least one method.

| strain name | NAuRA profiles | PCR profiles | SE detection in food | Discordance |
| --- | --- | --- | --- | --- |
| 05CEB01STA | *sea, sec, sel, selx, tsst-1* | *sea,sec* | NA |  |
| 05CEB18STA | *sea, seh, sek, seq, selx, tsst-1* | *sea,seh* | SEA |  |
| 05CEB52STA | *seb, seg, sei, sem, sen, seo, selu, selx, selz* | *seb,seg,sei* | NA |  |
| 07CEB132STA | *sed, selj, ser, selx* | *sed, selj, ser* | NA |  |
| 07CEB151STA | *selx, selz* | Negative | NA |  |
| 07CEB153STA | *sec, sel, selx* | *sec* | NA |  |
| 07CEB234STA | *selx, selz, tsst-1* | Negative | NA |  |
| 07CEB89STA | *sea, selx* | *sea* | SEA |  |
| 07CEB90STA | *sec, seg, sei, sel, sem, sen, seo, selu* | *sec,seg,sei* | NA |  |
| 07CEB91STA | *sep, selx* | Negative | NA | No detection of *sep* gene by PCR |
| 07CEB93STA | *selx* | Negative | NA |  |
| 07CEB94STA | *sea, seg, sei, sem, sen, selx* | *sea,seg,sei* | NA |  |
| 09CEB04STA | *selx* | Negative | NA |  |
| 09CEB204STA | *sea, selx* | *sea* | negative | No detection of SEA in food |
| 09CEB303STA | *selj, ser, ses, set, selx* | *selj,ser* | NA |  |
| 09CEB314STA | *see, selx* | *see* | SEE* |  |
| 09CEB319STA | *see, selx* | *see* | SEE* |  |
| 09CEB329STA | *see, selx* | *see* | SEE* |  |
| 11CEB145STA | *sea, sec, seg, sei, sel, sem, sen, seo, selx, tsst-1* | *sea,sec,seg,sei* | NA |  |
| 11CEB272STA | *sea,sed,selj,ser, selx* | *sea,sed,selj,ser* | NA |  |
| 11CEB273STA | *sed, selj, ser, selx* | *sed,selj,ser* | NA |  |
| 11CEB274STA | *selx, selz* | Negative | NA |  |
| 11CEB275STA | *selx, selz* | Negative | NA |  |
| 11CEB276STA | *sed, selj, ser, selx* | *sed,selj,ser* | NA |  |
| 11CEB277STA | *seg, sei, sem, sen, seo, selu, selx, sely* | *seg,sei* | NA |  |
| 11CEB278STA | *sed, selj, ser, selx* | *sed,selj,ser* | NA |  |
| 11CEB279STA | *seg, sei, sem, sen, seo, selu, selx, sely* | *seg,sei* | NA |  |
| 11CEB280STA | *selx, selz* | Negative | NA |  |
| 11CEB281STA | *sed, selj, ser, selx* | *sed,selj,ser* | NA |  |
| 11CEB282STA | *sea, sed, selj, ser, selx* | *sea,sed,selj,ser* | NA |  |
| 11CEB283STA | *selx, selz* | Negative | NA |  |
| 11CEB284STA | *sed, selj, ser, selx* | *sed,selj,ser* | NA |  |
| 11CEB285STA | *selx, selz, tsst-1* | Negative | NA |  |
| 13CEB175STA | *sea, sed, selj, ser, selx* | *sea,sed,selj,ser* | NA |  |
| 13CEB176STA | *seb, sek, seq, selx* | *seb* | NA |  |
| 13CEB177STA | *sec, seg, seh, sei, sel, sem, sen, selu, selx* | *sec,seg,seh,sei* | NA |  |
| 13CEB178STA | *sed, seg, sei, selj, sem, sen, seo, ser, selx* | *sed,seg,sei,selj,ser* | NA |  |
| 13CEB179STA | *sec, seg, sei, sel, sem, sen, seo, selu, selx* | *sec,seg,sei* | NA |  |
| 13CEB181STA | *sea, seg, seh, sei, sem, sen, seo, selu, tsst-1* | *sea,seg,seh,sei* | NA |  |
| 13CEB182STA | *sec, sei, sel, sen, seo, selu, selx, sely, selz, tsst-1* | *sec,seg,sei* | NA | No detection of *seg* gene by NAuRA |
| 13CEB184STA | *seh, selx* | *seh* | NA |  |
| 13CEB188STA | *seg, sei, sem, sen, seo, sep, selx* | *seg,sei,sep* | NA |  |
| 13CEB190STA | *sec, sei, sel, sen, seo, selu, selx, sely, selz, tsst-1* | *sec,seg,sei* | NA | No detection of *seg* gene by NAuRA |
| 13CEB191STA | *sed, seg, sei, selj, sem, sen, seo, sep, ser, selx* | *sed,seg,sei,selj,sep,ser* | NA |  |
| 13CEB193STA | *sea, sec, sel, selx, tsst-1* | *sea,sec* | SEA | No detection of SEC in food |
| 13CEB235STA | *sea, selx* | *sea* | NA |  |
| 13CEB239STA | *sea, selx* | *sea* | NA |  |
| 13CEB243STA | *sea, selx* | *sea* | NA |  |
| 13CEB251STA | *sec, sel,selx, tsst-1* | *sec* | NA |  |
| 13CEB257STA | *sec, sel, selx, tsst-1* | *sec* | NA |  |
| 13CEB307STA | *sea, sec, sek, sel, seq, selx* | *sea,sec* | SEA, SEC° |  |
| 13CEB308STA | *sea, selx* | *sea* | SEA° |  |
| 13CEB309STA | *sea, selx* | *sea* | SEA° |  |
| 13CEB310STA | *sea, sec, sek, sel, seq, selx* | *sea,sec* | SEA, SEC° |  |
| 13CEB311STA | *sep, selx* | *sep* | negative° |  |
| 13CEB312STA | *sea, seg, sei, sem, sen, seo,selu* | *sea,seg,sei* | SEA° |  |
| 13CEB313STA | *sea, seg, sei, sem, sen, seo,selu* | *sea,seg,sei* | SEA° |  |
| 13CEB314STA | *sea, seg, sei, sem, sen, seo,selu* | *sea,seg,sei* | SEA° |  |
| 13CEB315STA | *sea, seg, sei, sem, sen, seo,selu* | *sea,seg,sei* | SEA° |  |
| 13CEB316STA | *sea, seg, sei, sem, sen, seo,selu* | *sea,seg,sei* | SEA° |  |
| 13CEB317STA | *sea, seg, sei, sem, sen, seo,selu* | *sea,seg,sei* | SEA° |  |
| 13CEB318STA | *sea, seg, sei, sem, sen, seo,selu* | *sea,seg,sei* | SEA° |  |
| 13CEB319STA | *sea, seg, sei, selj, sem, sen, seo, ser, selx* | *sea,sed,seg,sei,selj,ser* | SEA° | No detection of *sed* gene by NAuRA |
| 13CEB320STA | *sea, seg, sei, selj, sem, sen, seo, ser, selx* | *sea,sed,seg,sei,selj,ser* | negative° | No detection of *sed* gene by NAuRA, and No detection of SEA in food |
| 13CEB322STA | *sea, seh, sek, seq, selx* | *sea,seh* | SEA° |  |
| 13CEB323STA | *sec, seg, sei, sel, sem, sen, seo, selu, selx* | *sec,seg,sei* | SEC° |  |
| 13CEB324STA | *sea, seh, sek, seq, selx* | *sea,seh* | SEA° |  |
| 13CEB326STA | *negative* | Negative | negative° |  |
| 13CEB327STA | *seg, seh, sei, sem, sen, seo, selu, tsst-1* | *seg,seh,sei* | negative° |  |
| 13CEB328STA | *seg, seh, sei, sem, sen, seo, selu, tsst-1* | *seg,seh,sei* | negative° |  |
| 13CEB329STA | *seg, sei, sem, sen, seo, selx* | *seg,sei* | negative° |  |
| 13CEB332STA | *sec, sel, selx, tsst-1* | *sec* | NA |  |
| 13CEB412STA | *sea, seh, sek, seq, selx, tsst-1* | *sea,seh* | negative | No detection of SEA in food |
| 13CEB417STA | *sea, seh, sek, seq, selx, tsst-1* | *sea,seh* | negative | No detection of SEA in food |
| 13CEB422STA | *sea, seh, sek, seq, selx, tsst-1* | *sea,seh* | negative | No detection of SEA in food |
| 13CEB427STA | *sea, seh, sek, seq, selx, tsst-1* | *sea,seh* | negative | No detection of SEA in food |
| 13CEB52STA | *selx* | Negative | NA |  |
| 14A | *sec, sed, seg, sei, selj, sel, sem, sen, seo, ser, selu* | *sec,sed,seg,sei,selj,ser* | NA |  |
| 15SBCL1201STA | *seb, sep, selx* | *seb,sep* | NA |  |
| 15SBCL1206STA | *seb, sep, selx* | *seb,sec,sep* | NA | No detection of *sec* gene by NAuRA |
| 15SBCL1211STA | *selx* | Negative | NA |  |
| 15SBCL1218STA | *sep, selx* | *sep* | NA |  |
| 15SBCL1220STA | *selx* | Negative | NA |  |
| 15SBCL1228STA | *seb, sep, selx* | *seb,sep* | NA |  |
| 15SBCL1251STA | *seb, sep, selx* | *seb,sep* | NA |  |
| 15SBCL1261STA | *selx* | Negative | NA |  |
| 15SBCL1262STA | *seb, sep, selx* | *seb,sep* | NA |  |
| 15SBCL1267STA | *seb, sep, selx* | *seb,sep* | NA |  |
| 15SBCL1292STA | *seg, sei, sem, sen, seo, selu, selx, sely* | *seg,sei* | negative |  |
| 15SBCL1299STA | *seg, sei, sem, sen , seo, selu, selx, sely* | *seg,sei* | negative |  |
| 15SBCL1314STA | *sea, sei, sem, sen, seo, selu, tsst-1* | *sea,seg,sei* | SEA | No detection of *seg* gene by NAuRA |
| 15SBCL1391STA | *selx* | Negative | NA |  |
| 15SBCL1397STA | *seg, sei, sem, sen, seo, selu, selx, sely* | *seg,sei* | NA |  |
| 15SBCL1404STA | *sec, sel, selx, tsst-1* | *sec* | NA |  |
| 15SBCL1409STA | *seg, sei, sem, sen, se, selu, selx, sely* | *seg,sei* | NA |  |
| 15SBCL1428STA | *seg, sei, sem, sen, seo, selu, selx, sely* | *seg,sei* | NA |  |
| 15SBCL1430STA | *seg, sei, sem, sen, seo, selu, selx, sely* | *seg,sei* | negative |  |
| 15SBCL1434STA | *selx, selz* | Negative | NA |  |
| 15SBCL1438STA | *sec, sei, sel, sen, seo, selu, selx, sely, selz, tsst-1* | *sec,seg,sei* | NA | No detection of *seg* gene by NAuRA |
| 15SBCL1506STA | *sea, sek, seq, selx* | *sea* | NA |  |
| 15SBCL1507STA | *seg, sei, sem, sen, seo, sep, selx* | *seg,sei,sep* | NA |  |
| 15SBCL1509STA | *sep, selx* | *sep* | NA |  |
| 15SBCL1517STA | *sec, seg, sei, sel, sem, sen, seo, selu, selx* | *sec,seg,sei* | NA |  |
| 15SBCL1520STA | *sea, seh, selx* | *sea,seh* | SEA |  |
| 15SBCL1527STA | *seg, sei, sem, sen, seo, selu, selx* | *seg,sei* | SEA |  |
| 15SBCL1540STA | *sea, seh, selx* | *sea,seh* | SEA |  |
| 15SBCL1548STA | *sea, seh, selx* | *sea,seh* | SEA |  |
| 15SBCL1550STA | *seg, sei, sem, sen, seo, sep, selx* | *seg,sei,sep* | SEA |  |
| 15SBCL1560STA | *sea, seh, sek, seq, selx* | *sea,seh* | NA |  |
| 15SBCL1565STA | *sea, seh, sek, seq, selx* | *sea,seh* | SEA |  |
| 15SBCL1570STA | *sea, seh, sek, seq, selx* | *sea,seh* | NA |  |
| 15SBCL1571STA | *sea, seh, sek, seq, selx* | *sea,seh* | NA |  |
| 15SBCL1576STA | *sea, seh, sek, seq, selx* | *sea,seh* | negative |  |
| 17SBCL03STA | *selx* | Negative | NA |  |
| 17SBCL07STA | *selx* | Negative | NA |  |
| 17SBCL08STA | *seg, sei, sem, sen, seo, selx* | *seg,sei* | NA |  |
| 17SBCL09STA | *seg, sei, sem, sen, seo, selx* | *seg,sei* | NA |  |
| 17SBCL13STA | *sea, seg, seh, sei, sek, sem, sen, seo, seq, selx, tsst-1* | *seg,sei* | NA | No detection of *sea* and *seh* genes by NAuRA |
| 17SBCL18STA | *selx, sely* | Negative | NA |  |
| 17SBCL202STA | *seg, sei, sem, sen, seo, selu, selx, sely* | *seg,sei* | negative |  |
| 17SBCL208STA | *seg, sei, sem, sen, seo, selu, selx, sely* | *seg,sei* | negative |  |
| 17SBCL214STA | *seg, sei, sem, sen, seo, selu, selx, sely* | *seg,sei* | negative |  |
| 17SBCL220STA | *seg, sei, sem, sen, seo, selu, selx, sely* | *seg,sei* | negative |  |
| 17SBCL223STA | *selx* | Negative | negative |  |
| 17SBCL225STA | *seg, sei, sem, sen, seo, selu, selx, sely* | *seg,sei* | negative |  |
| 17SBCL532STA | *seh, selx* | *seh* | NA |  |
| 17SBCL533STA | *seh, selx* | *seh* | NA |  |
| 17SBCL580STA | *sea, sed, seg, sei, selj, sem, sen, seo, ser, selx, tsst-1* | *sea,sed,seg,sei,selj,ser* | SEA, SED |  |
| 17SBCL585STA | *sea, sed, seg, sei, selj, sem, sen, seo, ser, selx, tsst-1* | *sea,sed,seg,sei,selj,ser* | SEA, SEC, SED | No detection of *sec* gene by NAuRA and PCR |
| 17SBCL586STA | *sea, sed, seg, sei, selj, sem, sen, seo, ser, selx, tsst-1* | *sea,sed,seg,sei,selj,ser* | SEA, SEC, SED | No detection of *sec* gene by NAuRA and PCR |
| 17SBCL778STA | *selx* | Negative | negative |  |
| 1A | *seg, sei, sem, sen, seo, sep, selx* | *seg,sei,sep* | NA |  |
| 294E | *sea, sed, selj, ser, selx* | *sea,sed,selj,ser* | NA |  |
| 333E | *selx* | Negative | NA |  |
| 367F | *seg, sei, sem, sen, seo, sep, selx* | *seg,sei,sep* | NA |  |
| 36A | *seh, selx* | *seh* | NA |  |
| 388F | *sea, selx* | *sea* | SEA |  |
| 42A | *sea, seb, sek, seq, selx* | *sea,seb* | NA |  |
| 43A | *sec, seg, seh, sei, sel, sem, sen, seo, selu, selx* | *sec,seg,seh,sei* | NA |  |
| 44A | *sec, sek, sep, seq, selx, selz* | *sec,sep* | NA |  |
| 45A | *sed, selj, ser, selx* | *sed,selj,ser* | NA |  |
| 46A | *see, seq, selz* | *see* | NA |  |
| 47A | *sek, seq, selx, tsst-1* | Negative | NA |  |

NA : Not Available

*Results from Roussel et al. (2015)

°Results from Denayer et al. (2017)
